# Dynamic Surface Chemistry of Catalysts in Oxygen Evolution Reaction

**DOI:** 10.1002/smsc.202100011

**Published:** 2021-05-09

**Authors:** Zongkui Kou, Xin Li, Lei Zhang, Wenjie Zang, Xiaorui Gao, John Wang

**Affiliations:** ^1^ Department of Materials Science and Engineering National University of Singapore 117574 Singapore Singapore; ^2^ Jiangsu Laboratory of Advanced Functional Materials School of Electronic and Information Engineering Changshu Institute of Technology Changshu 215500 P. R. China

**Keywords:** computational simulations, dynamic surface chemistry, in situ and operando characterization, oxygen evolution reactions, surface reconstruction

## Abstract

Electrocatalytic oxygen evolution reaction (OER) is a crucial anode reaction where electrocatalysts are the key elements and their dynamic surface chemistry runs throughout the entire process. Herein, we examine the latest advances and challenges in understanding of the dynamic surface chemistry of OER electrocatalysts. There are electrochemical origin and driving force for the dynamic surface nature, where several processes can take place either concurrently or sequentially, including reconstruction (i.e., phase formation/transformation, morphological change, and compositional change), vacancy generation and filling/refilling, and the intermediate adsorption–desorption process on catalytic surface. These dynamic surface processes of OER catalysts are impacted by not only the reaction and service conditions, including the (local) pH and its gradient distribution, applied potential, types and concentration of exotic ions and external fields on top of the nature of catalysts/precatalysts, but also their interactions. Due to the local, time‐dependent and instant nature, there are considerable challenges in tracing, modelling and understanding of the complete dynamic surface chemistry of catalysts in OER, by means of ex situ, in situ and operando experimental investigations. Therefore, computational studies and dynamic simulations help provide key insights in future pursuits, where there is critical need for a multiscale computational modelling approach encompassing all these aspects.

## Graphical abstract

Dynamic surface chemistry of catalysts dominates the actual oxygen evolution reaction (OER) process and performance. It involves either the reversible or irreversible dynamics, or both. There are strong effects of electrochemical servicing environments and external fields on OER. Both operando experiments and theoretical insights are particularly important.

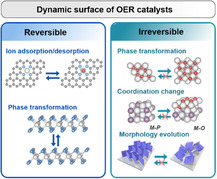

## 1 Introduction

With the ever rising depeletion of nonrenewable energy sources and the increasingly serious problem of environmental pollution in assoication, there is an apparent urgency to develop sustainable energy, where energy conversion and storage are among the key technologies. Currently, there are several key electrochemcial energy conversion systems, such as water splitting, fuel cells, dinitrogen/carbon dioxide fixation, and metal–air batteries, that have been widely studied.^[^ ^1^ ^]^ To enable these technologies, electrocatalytic oxygen evolution reaction (OER) is the core anode reaction, where it governs the overall performance, efficiency, and stability.^[^ ^2^ ^]^

**Figure 1:**
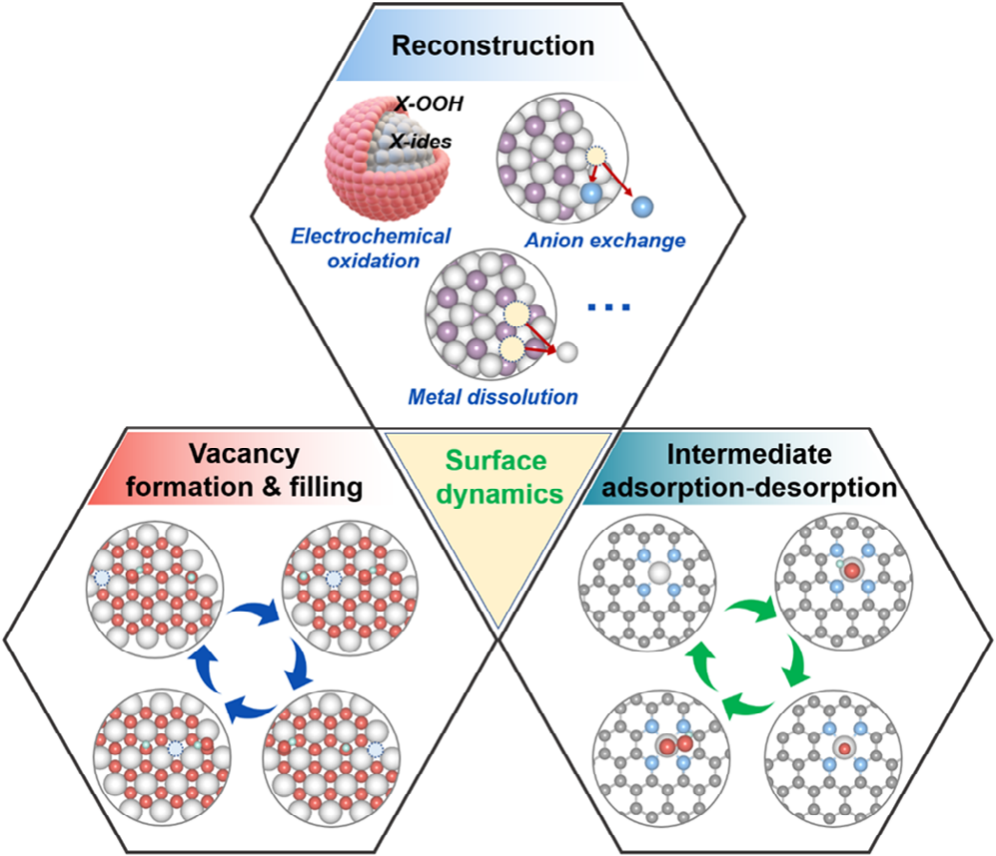
An overview of the schematic surface dynamics on OER catalyst surfaces.

In the OER process, molecular oxygen is produced by the oxygen–oxygen bond formation, accompanying with four proton‐coupled electron transfer steps.^[^ ^3^ ^]^ The accumulation of energy at each step makes OER kinetics largely sluggish and results in a large overpotential. Therefore, exploring high‐efficiency electrocatalysts is apparently the key to overcome the large energy barrier and reduce the overpotential to minimize the energy loss ingerent. To date, several noble metal‐based electrocataysts, typically such as RuO_2_^[^ ^4^ ^]^ and IrO_2_,^[^ ^5^ ^]^ have been extensively studied and are regarded as the most efficient OER electrocatalysts due to their low overpotentials. However, the scarcity, expensiveness, and instability in both acidic and alkaline electrolytes under high anodic potentials are limiting their wide applications in large‐scale energy conversion devices.^[^ ^6^ ^]^ Therefore, considerable research is now transferred to reducing the loading amount of these noble metals, and more interestingly investigating the alternative low‐cost and earth‐abundant transition metals, where the OER electrocatalysts are in different formulas of transition metal compounds, such as oxides,^[^ ^7^ ^]^ hydroxides,^[^ ^8^ ^]^ borides,^[^ ^9^ ^]^ carbides,^[^ ^10^ ^]^ nitrides,^[^ ^11^ ^]^ chalcogenides,^[^ ^12^ ^]^ etc. Due to the relatively reactive nature of these transition metal compounds at high OER potentials, they will undergo steady changes on surfaces/subsurfaces under the reaction conditions, which are considerably different from conditions under which they were synthesized. Therefore, they work actually as the “precatalysts,” rather than the authentic catalysts. In the OER process, they undergo a series of drastic reconstruction including phase formation and transformation, morphological and compositional changes under an oxidizing potential, evetually leading to the formation of self‐assembled amorphous metal oxides/(oxy)hydroxides active layers on the surface of precatalysts in alkaline electrocatalytic envioronment.^[^ ^13^ ^]^ These new species of metal oxides/(oxy)hydroxides are among the active players. Compared with the common crystalline metal oxides/(oxy)hydroxide, these reconstruction‐derived counterparts possess more oxygen vacancies, which can help tune the interactions between surficial sites and oxygen intermediates, promoting a higher electrocatalytic activity.^[^ ^14^ ^]^ Therefore, a thorough understanding on the actual reconstruction, which is the key part of the dynamic surface chemistry, is vitally important in design and development of efficient OER electrocatalysts (**Figure** **1**).

The reconstruction reaction is of dynamic, adaptive, potential‐dependent, precatalyst‐determining (static but synthetic processing‐determining) features.^[^ ^15^ ^]^ The surface evolution and degree of reconstruction are initially dependent on the composition, phase, morphology, and structure of the precatalysts, and then strongly impacted by the reaction and servicing conditions in OER as well as their interaction. The (sub)surface atoms on the precatalysts will largely determine the potential and depth of surface reconstruction. For example, Ni and Co possess different oxidation potentials prior to OER, respectively.[^11^, ^16^] The unstable surfaces can directly result in deep phase transformation into active metastable/stable phases.^[^ ^17^ ^]^ The structure and morphology of the precatalyst can also affect the evolution and degree of reconstruction, where, for example, the size effect can lead to faster rate and more depth of reconstruction at the smaller sizes of catalyst species.^[^ ^18^ ^]^ Upon initiation of the reconstruction, the process will continue and more interestingly it can vary, as a result of the changing environment where the reaction and servicing conditions play a determining role. For example, different alkaline ions will produce a different level and rate of surface reconstruction, according to the subsequence of Li < Na < K < Rb < Cs.^[^ ^19^ ^]^

The surface atoms at the electrode (electrocatalyst)/electrolyte interface under the servicing condition are dynamically changed in their structural integrity. The dissolution of metal ions along with the applied potential inevitably creates numerous vacancies, and these vacancies are filled by cations/anions in the electrolyte under the same situations (Figure 1).^[^ ^20^ ^]^ Specially, oxygen vacancy dynamics have been widely demonstrated to probably involve in the catalytic OER process.^[^ ^21^ ^]^ Therefore, at a given potential, vacancy formation and filling/refilling can reach an equilibrium, unless the surface reconstruction is rapidly ongoing and the catalyst degradation is in progress. In particular, for a classic lattice oxygen involving in the OER process, an understanding on the vacancy dynamics will be important in tracing the activation and optimizing the performance of an OER catalyst.^[^ ^22^ ^]^ Indeed, vacancy dynamics have been considered as a key component in the dynamic surface chemistry of OER catalysts.

To understand the dynamic surface chemistry of catalysts in OER, various ex situ, in situ, and operando characterization methods have been used in attempts to unveil the actual process, and to identify the active sites and explore the electrocatalytic mechanism of OER. In general, it is rather challenged to monitor the actual dynamic surface chemistry, although the composition, phase, structure, and morphology can be real‐time recorded in the OER process.^[^ ^21^, ^23^ ^]^ Therefore, there are considerably ongoing efforts in developing new and high‐resolution in situ and operando characterization techniques to analyze and identify the catalytic active sites and reaction mechanisms. In addition, the intermediates (i.e., *O, *OH, and *OOH) are dynamically adsorbed and desorbed on the catalytic sites in the OER process (Figure 1). As key components interacting with the OER dynamic surface, they can be monitored by various in situ and operando characterization techniques for unlocking the actual catalytic mechanism.^[^ ^24^ ^]^ A number of previous computation simulations have been devoted to identifying these intermediates‐related configurations and energy barriers.^[^ ^25^ ^]^ It is, however, noted that the surface reconstruction and the impacting factors of reaction and servicing conditions were not fully considered in some of these mechanism understandings, although they are important in deciding the dynamic surface chemistry and actual catalytic process.

**Figure 2:**
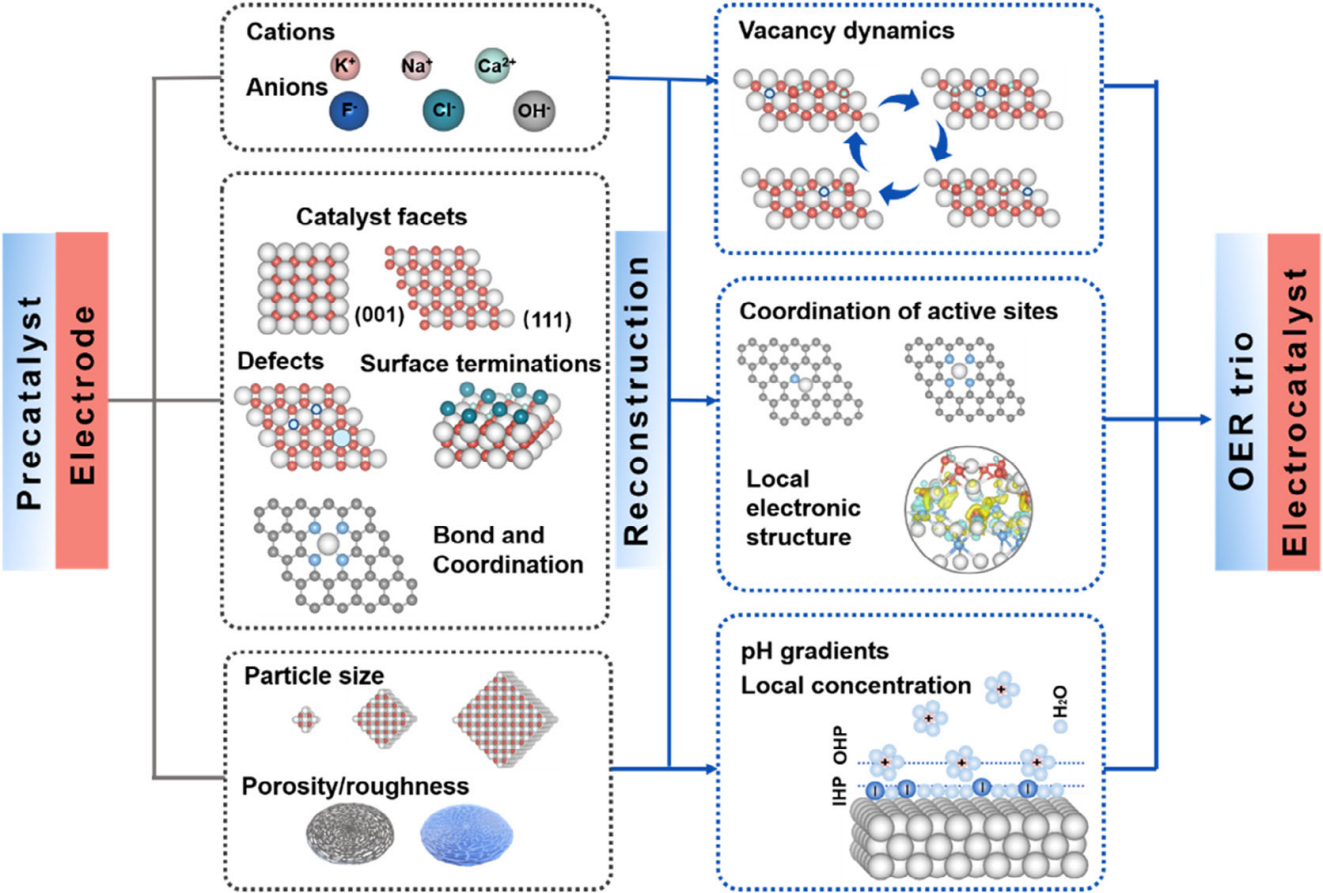
An overview of themes discussed in this review: schematic of the different length scales involved in the understanding of dynamic surface chemistry and their relevant parameters.

In this review, we provide in‐depth insights into recent advances and challenges in understanding of the dynamic surface chemistry of electrocatalysts in OER process, which will be disclosed and discussed in depth. An overview of some of the themes covered in this review is shown in **Figure** **2**. The fundamental origin of surface dynamics and reconstruction of electrocatalysts is introduced. The surface vacancy dynamics involved in the surface self‐reconstruction and the subsequent OER is illustrated. The influence factors including reaction and servicing conditions, electrocatalyst morphology, and surface/subsurface atom configurations are discussed in detail. Indeed, a thorough understanding on the reconstruction process will help bridge the precatalysts with the true catalysts in the OER process, such that the relationship between them will be further explored. We will also examine the experimental investigations into the dynamic surface chemistry, which are typically done by the advanced in situ and/or operando characterization techniques, for instance by resonance Raman spectroscopy, X‐ray absorption spectroscopy (XAS), Fourier‐transform infrared spectroscopy (FT–IR), ambient pressure X‐ray photoelectron spectroscopy (APXPS), differential electrochemical mass spectrometry (DEMS), and etc. They help track down the reconstruction, capture dynamic surface chemistry, analyze real‐time reaction products, and establish the rather deep mechanistic insights.^[^ ^1^, ^26^ ^]^ With the comprehensive theoretical calculations based on the density functional theory (DFT) and/or molecule dynamics, the enhancement or degradation mechanism of catalytic activities can be clarified.[^26^, ^27^] The theoretical calculations are normally used to analyze the Gibbs free energy of each of the intermediate steps, thus determine which steps are more plausible. Based on the recent advances, the challenges and future perspectives are put forward. With the knowledge on the dynamic surface chemistry and the actual self‐reconstruction process of OER electrocatalysts, new electrocatalysts with superior OER performance can then be feasibly designed.

## 2 Origins of Electrochemical Reconstruction

**Figure 3:**
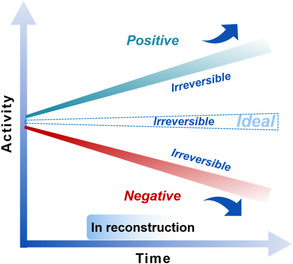
Reconstruction effect on OER activity as a function of reconstruction time.

**Figure 4:**
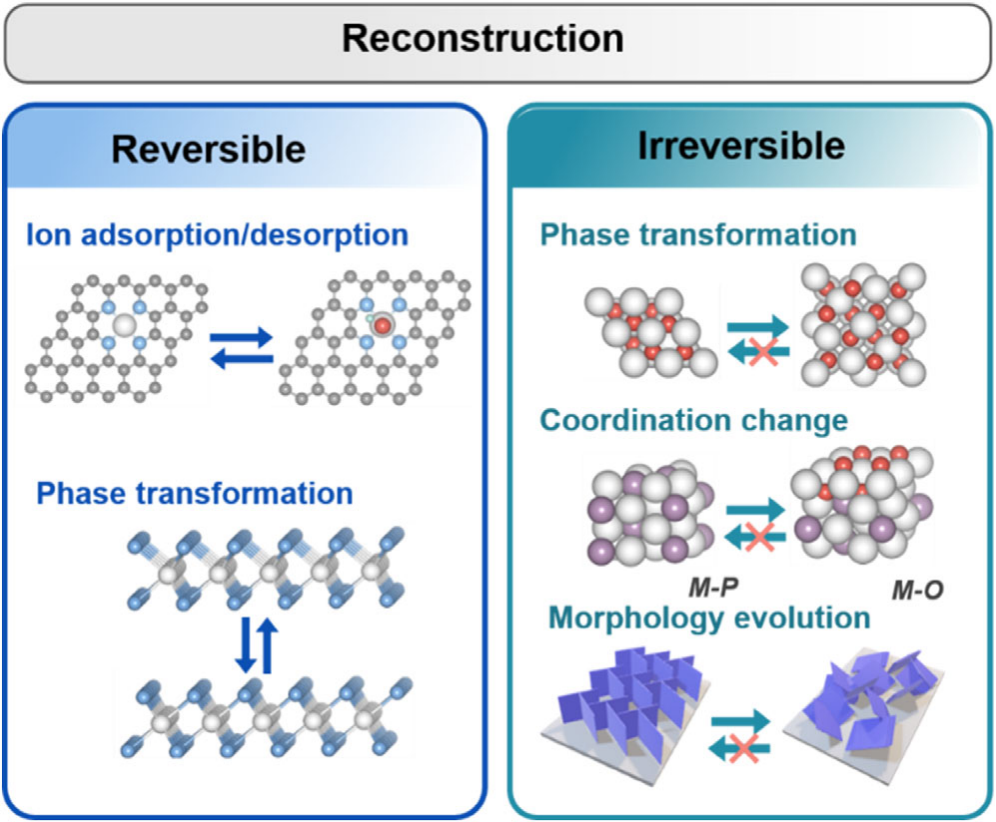
An overview of reversible or irreversible reconstruction processes.

The OER process commonly proceeds at a high positive potential of more than 1.4 V.^[17b]^ At such high potential, (sub)surface atoms on an electrocatalyst tend to be oxidized. The surficial sites are therefore dynamic in nature, driving in situ changes and reconstruction. Therefore, the reconstruction typically refers to the changes of an electrocatalyst in the catalytic process under servicing environment, which generates considerable differences from the initial electrocatalyst in terms of the composition, phase(s), crystallinity, and structures at various scales, mainly on the surfaces/subsurfaces. Electrochemical reconstruction is a potential‐dependent and adaptive electrochemical reaction, and generally more active than OER at the given positive potential. The thus generated reconstruction can lead to the variation of surface chemistry in different ways, including composition, phase, and structure. It is well known that the electrocatalytic reactions occur on the surface of electrocatalysts. Therefore, the reconstruction process will undoubtedly affect the time‐dependent surface states of electrocatalysts, thus leading to the variation of the electrocatalytic performance.^[^ ^28^ ^]^ An ideal electrocatalyst will have its OER activity remaining largely unchanged along with the servicing time, by possessing a good structural and performance stability (**Figure** **3**). However, for most of the electrocatalysts, there is a steady reconstruction taking place as soon as it is placed under the servicing environment, where the reconstruction can be either positive or negative, in terms of affecting the OER activity over time. The reconstruction involves changes in one or more forms of the composition, phase(s), micro/nanostructures, and crystallinity, at the surface/subsurface of the precatalyst. These reconstruction features can be reversible or irreversible along the reaction and servicing conditions (**Figure** **4**).[^15^, ^29^] Among the reversible reconstructions is the actual catalytic process involving the intermediate absorption and desorption, where there is the charging process of catalyst surface at the increasing potential and a discharging process along the reductive potential. The irreversible reconstructions typically include the unrecovered processes of new phase formation, phase transformation, morphological change, and irreversible intermediate desorption (e.g., those originated from strong chemical adsorption of intermediates on the catalytic surfaces). They all are deeply impacted by the reaction and servicing conditions, as well as the composition, morphology, phase, and structure of the precatalysts. Therefore, a proper study of reconstruction chemistry during OER is very valuable for deep understanding of the actual electrocatalytic process and mechanism, and developing of advanced electrocatalysts for OER.

The rate and degree of reconstruction are related to both electrocatalyst nature and external factors in servicing environment. The servicing conditions, such as the (local) pH value and gradient, type and nature of electrolyte, temperature and pressure, could all adjust the oxidation potentials and kinetics, therefore leading to multiple oxidation products.^[17b]^ For example, it has been suggested that a high pH can facilitate reconstruction and produce lower potentials as pH value increases on the reversible hydrogen electrode (RHE) scale.^[^ ^30^ ^]^ The surface oxidation of noble metal‐based electrocatalysts have been noted early in the last century.^[^ ^31^ ^]^ Taking RuO_2_ as an example, the oxidation peak before OER shifts toward lower potentials and OER activity enhances as the pH value of electrolyte increases, showing the pH‐dependent Ru oxidation and OER activity.^[^ ^32^ ^]^ The bulk pH can also affect the RuO_2_/water interface,^[^ ^33^ ^]^ and the interface between RuO_2_ and water containing many polarizable molecules and adsorbates such as water, O*, and OH*. The differences in water layer geometries and the formal valences of surface Ru result in a pH‐dependent behavior of the OER, and therefore affect the overall OER activity. The pH‐dependent reconstruction effects on OER behaviors have also been observed in the transition metal oxide‐based electrocatalysts, which are more reactive, such as iridium oxide (≈1 V vs RHE),^[^ ^34^ ^]^ rhodium oxide (≈1.4 V vs RHE),^[^ ^35^ ^]^ iron oxide (≈0.3 V vs RHE),^[^ ^35^ ^]^ nickel oxide (≈1.35 V vs RHE),^[^ ^36^ ^]^ cobalt oxide,^[^ ^37^ ^]^ and Fe‐containing Ni‐based oxides.^[^ ^38^ ^]^

The ability to sustain unattenuated performance under challenging service conditions entailing a combination of high corrosivity of the electrolyte (>1 m KOH), high‐temperature (>80 °C), and high current densities (>500 mA cm^−2^) is the ultimate criterion for practical viability.^[^ ^39^ ^]^ Therefore, the stability and OER activity of electrocatalysts in the electrolyte at high temperature and concentrations are very important for their practical application in industry. Andronescu and co‐authors have reported the influence of the temperature and electrolyte concentration on the structure and catalytic oxygen evolution activity of NiFe layered double hydroxide (NiFe LDH).^[^ ^39^ ^]^ The immersion experiment of NiFe LDH in the 30 wt%  KOH at 80 °C for 60 h revealed a transformation of the structure into a mixture of crystalline β‐Ni(OH)_2_ and discrete predominantly amorphous FeOOH containing minor nonhomogeneously distributed crystalline domains. These structural and compositional changes resulted in a drastic loss of the OER activity. Therefore, how to retain high OER performance including activity and stability needs to be further studied at such industrially relevant conditions.

In addition to the external factors in servicing environments, the nature of electrocatalyst including the composition, phase, facet, and structure can all affect the onset potential and rate of surface reconstruction. The different metal atoms are oxidized at the different potentials for generating oxidation products. For example, Ru(III), Ru(IV), and Ru(V) are present at ≈0.4, 1.0, and 1.4 V versus RHE, respectively.^[^ ^33^ ^]^ However, higher‐valence Ru such as RuO_4_ formed on the surface at 1.45 V versus RHE is unstable to exist for a long period of time during OER in both acidic and alkaline conditions, due to the easy dissolution or quick regeneration to lower valence state Ru ions.^[^ ^40^ ^]^ Compared to noble metal‐based compounds, the transition metal‐based electrocatalysts exhibit more stable oxidation product layers generated from the reconstruction. The main valence of surface metals varies based on the different transition metals. Taking Ni, Co, and Fe as examples, they are normally oxidized to Ni (III), Co (IV), and Fe (IV) during OER, corresponding to NiOOH, CoO_2_, and FeO_2_ in the alkaline condition, respectively. Normally, for Ni‐based electrocatalysts, the oxidation process proceeds with the first formation of α‐Ni(OH)_2_ phase, then the oxidization to γ‐NiOOH at about 1.35 V versus RHE, with Ni^IV^ (NiOO_2_) as the intermediate to O_2_ formation.^[^ ^38^ ^]^ But for β‐Ni(OH)_2_, the oxidation product is β‐NiOOH, which is easily converted to γ‐NiOOH with a higher average valence state under overcharging.^[^ ^41^ ^]^ The reconstruction of Ni‐based catalysts is normally reversible,^[11b]^ namely, the formed oxidation product could be reduced to initial valence state. However, the oxidized Co cations exhibit an irreversible surface reconstruction process. Fabbri et al. utilized operando X‐ray adsorption near‐edge structure (XANES) spectra to detect the current variation and shift of the Co K‐edge position.^[15a]^ They have observed that Co K‐edge shifts toward higher energy as the potential rises to 1.425 V (vs RHE), indicating that Co has been oxidized. A further oxidation reaction proceeds with the increase in potential to 1.55 V (vs RHE). However, when the potential reduces from 1.55 V (vs RHE) to 1.2 V (vs RHE), Co K‐edge has no significant changes, suggesting that the oxidation of the Co cations is mostly an irreversible process during OER. For the perovskite and spinel electrocatalysts, a dissolution of metal ions and lattice oxygen oxidation during OER often take place accompanying with the reconstruction process.[^15^, ^17^]

It is worth mentioning that even if certain precatalysts are congeneric, they often exhibit a diverse self‐reconstruction phenomenon, and the catalytic performance can be associated with the different structure features at nano and/or mesoscale. Those structures and morphologies with a large active surface area, such as hollow structure, core–shell nanostructure, 2D thin nanosheets, tend to quickly produce surface active species via the in situ electrochemical reconstruction. For example, a core–shell nanostructure can inherit the advantages from each components and their interfaces, and therefore more active sites.^[^ ^42^ ^]^ In contrast, at the interface between the two components there is facilitation of accelerating the electron transfer and thus leading to the redistribution of electrons on the catalyst surface and optimizing the surface chemical adsorption/desorption during OER.^[^ ^43^ ^]^ The reconstruction with a core–shell nanostructure usually happens on the surface shell layer within several tenths of a nanometer.^[11b]^ Therefore, the thus formed active oxide/(oxy)hydroxide layers are very thin, which then facilitates the intimate contact with the core material and induces the synergistic effect between them. In addition, some unstable surfaces can directly lead to an in deep phase transformation into active metastable/stable phases. For example, Ryu and co‐workers reported the in situ transformation of CoP in the alkaline electrolyte, which had experienced unique metamorphosis upon anodic potential cycling.^[^ ^44^ ^]^ The original rod‐shaped CoP nanoparticles were changed into a porous and nanoweb‐like architecture, even after just one cyclic voltammogram (CV) cycle. The substantial transition is believed to proceed through an oxidative process prior to the onset of oxygen evolution. Generally, the reconstruction dynamics can be described by electrochemical equilibrium potential theory: *G* = *G*_catalysts_‐*G*_precatalysts_ (*G* is the Gibbs free energy), which can be used to simply judge the energy barrier of surface reconstruction on different catalyst surfaces under the same reaction conditions. These values are directly related to some of the factors mentioned earlier (e.g., type of phase, atomic nature, morphology, structure of catalysts, and the external factors under the servicing condition).

## 3 Dynamics of Vacancy on Surface

It has been widely acknowledged that vacancy can be generated in the synthetic processing, reconstruction, or catalytic reactions of catalysts. For synthetic process, in general, vacancy is purposely designed for improving the catalytic performance by tuning the electronic structure of active sites.^[^ ^45^ ^]^ Moreover, a flexible surface/subsurface structure with a large concentration of oxygen vacancies favors the in situ dynamic reconstruction into a highly OER‐active (oxy)hydroxide surface layer.^[15a]^ For example, in perovskite oxides, the surface O vacancy plays as the active center with negligible structure change, correlated to the low structure flexibility.^[17b]^ The reconstruction can also in turn produce the vacancies on the catalyst surfaces, furtherly accelerating the reconstruction process and improving the catalytic performance.^[^ ^21^, ^46^ ^]^ In addition, during catalytic reactions, unavoidable metal ion dissolution can also create numerous vacancies in the catalysts.^[^ ^47^ ^]^ Sometimes, these vacancies can be filled by the ions in the electrolyte, resulting in a positive role on the catalytic mechanism and performance.^[^ ^37^ ^]^ Therefore, vacancy dynamics run through the overall OER process and determine all of activity, selectivity, and stability as well as even catalytic mechanisms, therefore attracting numerous attentions in the OER investigations.

**Figure 5:**
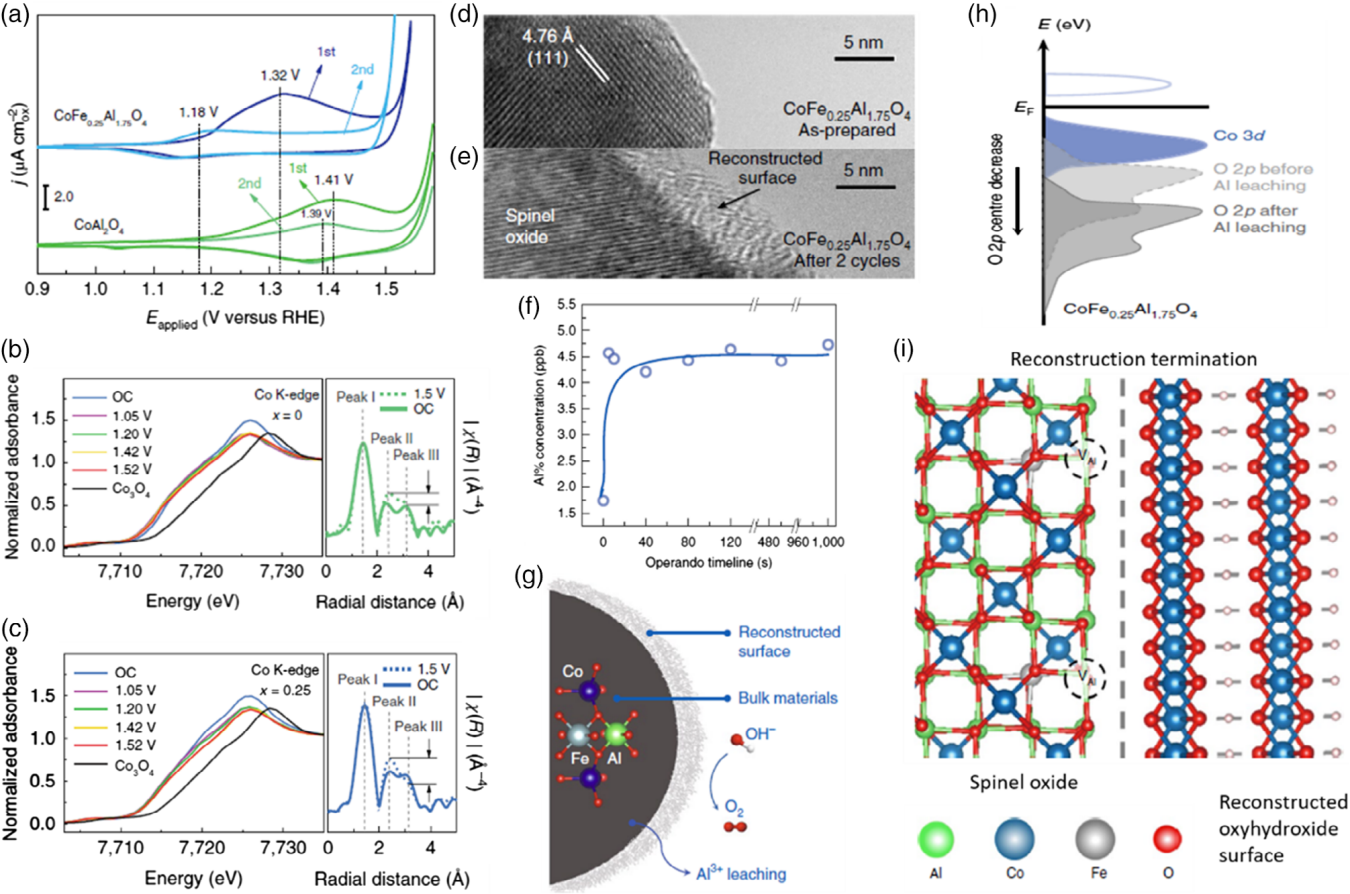
a) Pseudocapacitive behavior in the first and second cycles of CoFe_0.25_Al_1.75_O_4_ and CoAl_2_O_4_ during CV cycling. Normalized in situ Co K‐edge XANES analysis (left axis) under 1.05, 1.20, 1.42, and 1.52 V (vs RHE) with Co_3_O_4_ (Sigma Aldrich) as reference, as well as the in situ FT *k* ^3^χ(*R*) Co K‐edge EXAFS (right axis) under open‐circuit (OC) and 1.5 V (vs RHE): b) CoAl_2_O_4_ and c) CoFe_0.25_Al_1.75_O_4_. d) HRTEM images of the as‐prepared CoFe_0.25_Al_1.75_O_4_ and e) CoFe_0.25_Al_1.75_O_4_ after two cycles. f) ICP–MS test of the electrolyte for CoFe_0.25_Al_1.75_O_4_ cycling under an operation time of 0–1000 s (in 1 m KOH under 20 μA cm_ox_^−2^). g) Schematic of Al^3+^ leaching along with surface reconstruction of the spinel oxide. h) Schematic band diagrams of CoFe_0.25_Al_1.75_O_4_ with and without Al^3+^ vacancies. i) Schematic of CoFe_0.25_Al_1.75_O_4_, which terminates its surface reconstruction due to the termination of lattice oxygen oxidation. a–i) Reproduced with permission.^[^ ^46^ ^]^ Copyright 2019, Springer Nature.

Oxygen vacancies can be created by doping of heteroatoms in the precatalyst, the dissolution/leaching of cations or anions under the service condition, which will influence the surface charge distribution of electrocatalysts, therefore giving an opportunity to promote OER.^[^ ^48^ ^]^ For example, Wu et al studied the surface reconstruction dynamics of iron‐doped spinel CoAl_2_O_4_ for water oxidation.^[^ ^46^ ^]^ From the CV characterization as shown in **Figure** **5**a, one can see that the Fe‐doped CoAl_2_O_4_ (CoFe_0.25_Al_1.25_O_4_) exhibits a lower oxidation peak position (1.32 V vs RHE) than that of bare CoAl_2_O_4_ (1.41 V vs RHE), indicating a promoting effect of Fe on the preoxidation of Co (II) and facilitating the subsequent formation of Co oxyhydroxide. In addition, the pseudocapacitive charge from the first cycle is larger than that of the second cycle, suggesting that the surface reconstruction into oxyhydroxide might be an irreversible process. Therefore, it could be concluded that the thus formed Co oxyhydroxide active species on the surface of catalyst are relatively stable. The in situ X‐ray adsorption fine structure (XAFS) analysis shown in Figure 5b,c confirmed the active sites and the dynamic changes of Co sites during OER. The ratio of peak II to peak III in the CoFe_0.25_Al_1.25_O_4_ is higher than that in bare CoAl_2_O_4_ (peak II and III represent the radial distances of Co atoms in octahedral sites and in tetrahedral sites, respectively, to their neighboring metal atoms), revealing that a more steady surface reconstruction into Co oxyhydroxide in CoFe_0.25_Al_1.25_O_4_. Moreover, a higher increment of Co valency in CoFe_0.25_Al_1.25_O_4_ is observed in the pseudocapacitive region than that in the bare CoAl_2_O_4_, suggesting a more prevailed deprotonation process due to the Fe substitution. The surface‐reconstructed amorphous oxyhydroxide layer with several nanometers can be seen clearly from the high‐resolution transmission electron microscopy (HRTEM) images in Figure 5d. Meanwhile, there was partial dissolution of Al^3+^ ions in the alkaline electrolyte at the beginning of OER process as shown in Figure 5e, and then, it was quickly terminated when the operando timeline attained to 40 s. The dissolution of Al would tune the local electronic structure of the oxide to prevent further reconstruction (Figure 5f). The partial dissolution of Al^3+^ ions resulted in the creation of Al vacancies with the decrease in energy level of O 2p (Figure 5g), and activated the classic lattice oxygen oxidation involving OER process. Meanwhile, a large number of oxygen vacancies would be generated (Figure 5h) due to the structural flexibility resulted from the dissolution of Al^3+^. Therefore, the heteroatoms doping in the precatalyst and the dissolution of cations could modulate the dynamic surface structure and chemical properties, thus impacting the OER performance of catalysts.

There are other electrocatalysts that show a similar phenomenon of cation doping and leaching, enabling the fast surface reconstruction and ultimately enhancing the OER performance. For instance, CoNi doping in Fe_3_N induces the lattice expansion and modulates the electronic structure of Fe_3_N, helping optimize the adsorption of hydroxyl groups from the electrolyte in the OER process, and thus motivating the rapid and efficient surface reconstruction into CoNi–FeOOH on the Fe_3_N surface.^[14a]^ In addition, Ba_0.5_Sr_0.5_Co_0.8_Fe_0.2_O_3‐*δ*_ electrocatalyst exhibits a degree of leaching of the Ba^2+^ and Sr^2+^ ions to form a flexible perovskite structure with a large oxygen vacancy content, leading to a rapid surface evolution into an amorphous oxyhydroxide after a few cycles during OER.^[15a]^ In the case of La_1‐*x*_Sr_*x*_CoO_3*‐δ*_, the substitution of Sr^2+^ triggers the oxidation of lattice oxygen and up to 37 monolayers of oxides (≈14 nm) involved in the catalyst surface during the OER process.^[^ ^37^ ^]^ In another typical case, Duan et al. found the cation leaching of Cr in CoCr_2_O_4_ by activating the pristine material at high potential, which enabled the transformation of inactive spinel CoCr_2_O_4_ into a highly active catalyst.^[^ ^49^ ^]^ The depletion of Cr and consumption of lattice oxygen facilitate the formation of surface defects and oxygen vacancies, exposing Co species to reconstruct into active Co oxyhydroxides which differ from CoOOH.

**Figure 6:**
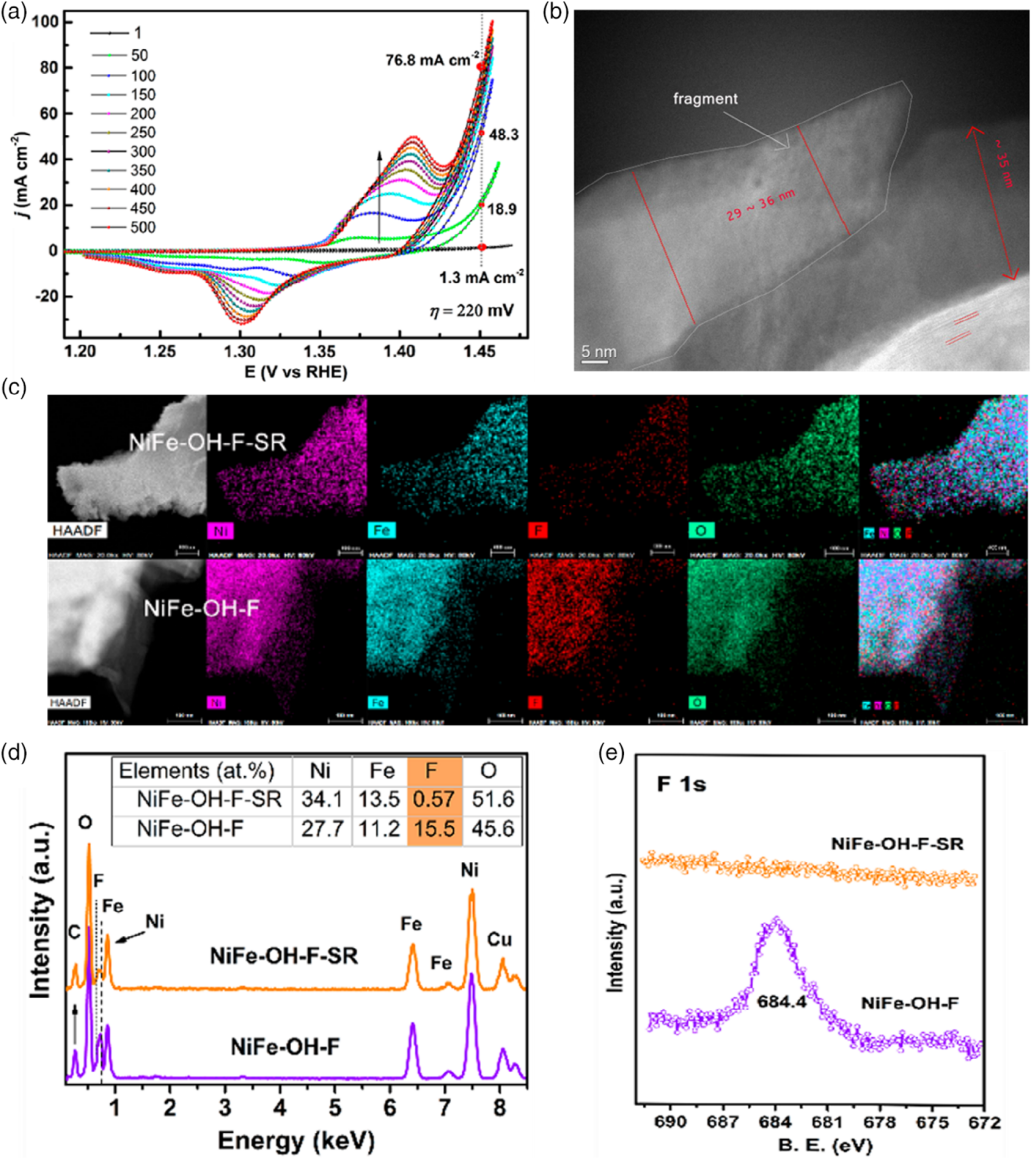
a) The entire 500 CV cycles for NiFe–OH–F. b) High angle annular dark field (HAADF)–STEM image of the postcycling NiFe–OH–F–SR. c,d) Elemental mapping images and EDX spectra of NiFe–OH–F–SR and NiFe–OH–F, respectively (the scale bars in the HAADF–STEM images of NiFe–OH–F–SR and NiFe–OH–F in panels (c) are 400 and 100 nm, respectively). e) High‐resolution XPS spectra of F 1 s. Reproduced with permission.^[^ ^76a^ ^]^ Copyright 2019, American Chemical Society.

In addition to cation vacancies, anion vacancies can be also produced during the OER process, thus facilitating surface reconstruction and producing unexpected efficient OER activity. For instance, Zhang et al. reported a fluoride (F^−^)‐incorporated NiFe hydroxide (NiFe–OH–F) nanosheet array.^[^ ^76a^ ^]^ As shown in **Figure** **6**a, OER current density increased rapidly before the 150th CV cycle and gradually gained stability after 400 cycles. The Ni oxidation peak area exhibited a dramatic increase and a negative shift of onset OER potential, indicating an increased amount of charge transfer and steady transformation into Ni oxyhydroxide. Upon CV cycling, a core–shell nanostructure with a thickness of ≈35 nm surface amorphous layer was observed in the scanning transmission electron microscopy (STEM) image as shown in Figure 6b. The energy‐dispersive X‐ray (EDX) elemental mapping and spectrum in Figure 6c showed that the elements of Ni, Fe, and O remained a rather uniform distribution after the surface reconstruction. However, a dramatic decrease in fluoride content was observed (Figure 6d), indicating that fluoride was dissolved during OER. The deduction was also verified by the X‐ray photoelectron spectroscopy (XPS) results in Figure 6e, where F 1s peak was almost disappeared, illustrating that the surface reconstruction under OER conditions might well be induced by fluoride leaching. As a basic feature of ion effect on surface reconstruction, other anions such as Se, S, N, B, and P have been also demonstrated to experience a respective degree of dissolution in the electrolyte under OER reaction.[^2^, ^6^, ^44^, ^51^]

As for the detection of vacancies, several characterizations, such as STEM, HRTEM, scanning tunneling electron microscopy (STM), XPS, electron paramagnetic spectroscopy (EPR), and XAS, have been used. For example, Gao et al. found the structural distortions (oxygen vacancies) by magnifying the HRTEM image.^[^ ^52^ ^]^ Vacancies can also be tracked by analyzing the intensity and shape of electron energy‐loss spectroscopy (EELS) edge, which reflects the underlying electronic structure.^[^ ^53^ ^]^ For example, Rong and coauthors investigated the lattice oxygen vacancies from CeO_2_ grown on Pt (111) by the STM.^[^ ^54^ ^]^ XPS has been a prominent tool in detecting the oxygen vacancies by effectively measuring oxygen vacancy peaks from surface oxygen‐deficient materials. Moreover, XPS measurement can obtain the information on concentration of oxygen vacancies. For example, Liu and co‐author used XPS characterization to determine a higher level of oxygen vacancies in Co_3_O_4_/CeO_2_ nanohybrids than those in bare Co_3_O_4_ and CeO_2_ by measuring the peak area of corresponding oxygen vacancy.^[^ ^55^ ^]^ Photoluminescence spectroscopy (PL) is also a useful characterization technique for studying electronic changes of materials and determining the degree of oxygen deficiency.^[^ ^56^ ^]^ The presence of oxygen vacancies generates new electronic states near the Fermi level and generally modulates the bulk electronics of a material. Peak position and intensity of the PL spectra can easily identify changes that occur upon such electronic modulations from oxygen vacancies. EPR technique can provide fingerprinting information of bulk and surface unpaired electrons, which is a strong indicator of oxygen vacancies. EPR signal intensity can be affected by the number of oxygen vacancies in materials. For example, Sun et al. found the EPR intensities were increased a lot after introducing oxygen vacancies in the materials.^[^ ^57^ ^]^ Raman spectroscopy is also an efficient tool in identifying oxygen‐deficient materials. Varying chemical bonds upon the induction of oxygen vacancies results in different vibrational modes. This change can produce lattice molecular vibrational level changes, which give rise to Raman shifts.^[^ ^58^ ^]^ XAS characterization is adopted to study the local geometric configurational changes and electronic structures of solids. For example, Xiao et al. used XAS technique to detect oxygen vacancies by probing the lower coordination number of cobalt.^[^ ^20^ ^]^ Considering every technique has its own restrictions, it is then sometimes unpersuasive to prove the existence of oxygen vacancy by using only one technique. For example, EPR cannot distinguish among the different types of defects (i. e., structural pits, cationic, and anionic vacancies). Therefore, different techniques shall be combined together to comprehensively analyze the existence and population of oxygen vacancies.

## 4 Correlation between Dynamic Surface Chemistry and Reaction/Servicing Conditions

Both reaction and servicing conditions play determining roles in the dynamic surface chemistry process. The charging of catalyst surfaces,^[^ ^59^ ^]^ applied potential,^[^ ^60^ ^]^ bulk and local pH,^[^ ^61^ ^]^ nature of the exotic ions,^[^ ^62^ ^]^ external fields,^[^ ^63^ ^]^ and mass‐transport conditions^[^ ^64^ ^]^ can all deeply influence the reconstruction process (Figure 2), and therefore largely determine the trio of OER (i.e., activity, step selectivity, and stability). Indeed, although there have been attempts of interpretating the individual effects in many studies reported, there is a general lack of systematic work due to the complication in association with the interaction of individual effects. In addition, given the importance of effects of the key reaction and servicing conditions on surface dynamics of OER catalysts, it is crucial to have proper standard protocols for meaningful evaluation of the OER trio. Therefore, not only the roles of reaction and processing conditions shall be deeply examined in this section but also the standard protocols are suggested accordingly in the end of article.

The degrees of surface reconstruction and vacancy formation/filling equilibria in the electrolyte are highly sensitive to the charging of catalyst surfaces, applied bias, pH, and electrolyte composition, which further determine different OER trio. The challenges in investigations into individual effects have resulted in some controversies or even wrong understanding about real active origins of OER on catalytic surfaces. Many have acknowledged, upon OER, that the surface composition of “true” catalyst in the metal X‐ides (X is C, N, P, S, Se, and Te and other nonoxygen elements) is metal oxide/(oxy)hydroxide,^[^ ^65^ ^]^ although a few studies have still simply stated that the X‐ide does not suffer from oxidation and remains stable even under rigorous oxidative conditions in the OER process.^[^ ^66^ ^]^ Indeed, the X‐ides are either completely oxidized, left unoxidized, or transformed into a core@shell structure upon OER. One of the most prominent conclusions has suggested that OER on the reconstruction surfaces of partially oxidized X‐ides is more active than the pure surficial materials (oxides/oxyhydroxides in situ formed on the surfaces of X‐ides).^[62b]^ A typical example of this effect happens on the Ni‐based/Co‐based electrocatalysts in which the OER activities of NiOOH and CoOOH reconstructed from Ni_3_N^[11b]^ and Co metal^[^ ^16^ ^]^ show the remarkable enhancement compared to their pure counterparts with similar micro–nanostructures as synthesized. The enhanced OER activities on the reconstructed surfaces have been proposed to be associated with high‐valent metal ions and/or vacancies (arising from a charging process of catalyst surfaces) in situ formed in the reconstructed oxides/oxyhydroxides shells,^[^ ^67^ ^]^ as well as the improved charge transfer in the X‐ides cores.[^11^, ^68^] These species act as active sites with higher intrinsic activity, leading to a sharp increase in the overall OER performance. A combined investigation of the operando DEMS and XAS has explored the OER dynamics and the individual Faradaic charge efficiencies of Ni–Fe oxyhydroxide electrocatalyst, and revealed that up to 75% of the Ni species have increased the oxidation state from +2 to +3 whereas the residual Ni is even oxidized into +4 during the OER process.^[^ ^69^ ^]^ The operando XANES analysis of oxygen vacancy (V_O_)‐rich Co_3_O_4_ during OER had further disclosed that the valence state of Co ions in V_O_–Co_3_O_4_ had a faster increase than that in the pure Co_3_O_4_, suggesting the vacancy‐induced coordination environment changes around active Co sites.^[^ ^21^ ^]^ More generally, a recent work has demonstrated that the formation of superoxol/peroxo‐like (O_2_)^n−^ species (i.e., NiOO* or CoOO*) during the OER process is crucial for enhancing OER activity.^[15b]^ The OER‐induced surface reconstruction builds an adaptive core–shell nanostructure, where NiOOH grows on delithiated LiNiO_2_ (delithiated‐LiNiO_2_/NiOOH). At this core–shell nanostructure, the lithium vacancies within the delithiated LiNiO_2_ core help optimize the electronic structure of the NiOOH shell to form stable NiOO* species, which enables better OER activity. DFT calculations have further revealed that deprotonated O sites are more energetically active than metallic Ni sites, but it could not be in agreement with a number of previous conclusions in which metallic Ni sites were always considered as active sites in the DFT investigations. Such difference in understanding of the active origins can well be associated with the dynamic OER interfaces between catalysts and reaction species, which are not still well imaged and probed using most of the techniques that have been developed.^[^ ^50^ ^]^ Therefore, a deep understanding would be required to address and hopefully unify the controversies.

The dynamic surface chemistry of OER catalysts gives rather complex electrochemical kinetics, which is rather challenging to be understood and controlled, and they are typically dependent on the applied potential exponentially (or called overpotential or bias). The charging of catalyst surface under a bias affects both bond formation and rupture of the reactive molecules, the effect of which on the electrocatalytic OER kinetics is often largely unknown. Very recently, the pulse voltammetry and operando XAS measurements were performed on iridium oxide to show that the applied bias cannot directly produce a substantial influence on the reaction coordinate, but affects the electrocatalytically generated current through charge accumulation on the catalyst surfaces.^[^ ^59^ ^]^ The activation free energy appears to drop down linearly with the amount of charging oxidation states under bias. This effect can be the fundamental origin of the electrocatalytic OER performance and can be evaluated using experimental measurement and computation studies. For example, in situ Raman spectroscopy and XAS tests have been widely performed to probe the effect of the applied potential on the surface reconstruction and vacancies dynamics of the OER catalysts.^[^ ^16^, ^21^, ^60^, ^70^ ^]^ A gradual transformation from pristine nickel‐based materials (NiNPS), to α‐Ni(OH)_2_/NiO, and then to active γ‐NiOOH has been found by Xiong et al to be potential dependent.^[^ ^71^ ^]^ Similar dynamic reconstruction processes were also observed by a number of recent observations.^[^ ^1^, ^15^, ^71^ ^]^ However, the γ‐NiOOH is not explicitly recovered when one applies a reduction potential, and the different dynamics of active sites accompanies by metal ion dissolution into the electrolyte under different bias, leading to a huge difficulty in the proper understanding of catalyst stability.

As soon as there is a change in the applied potential, the importance of the local pH and concentration of reaction species around the catalytic interfaces must be taken into account for comprehensive understanding of surface chemistry of catalysts during OER process. The overall analyses of current experiment data (especially the change of performance with CV) shall conclude the gradually increasing degree of reconstruction and the presence of gradual distribution of reaction species concentrations during OER process, which can well be resulted from the potential‐induced oxidation reaction and concentration difference. In any alkaline OER, a larger difference of local pH from the bulk pH will produce a faster reconstruction into oxyhydroxides of catalyst surfaces and therefore better OER activities.^[61a]^ One shall speculate that such a conclusion can be also used to explain why the exotic fields (i.e., electric and magnetic fields electric potentials, mechanic stress, etc.) have been reported to promote OER performance beyond the catalyst design.^[^ ^63^ ^]^ These exotic fields can remarkably change the local pH and local concentration around the catalytic interfaces by directing the charge transportation, therefore deeply impacting the dynamic surface chemistry of catalysts during OER process. Along this research direction, the importance of the reaction and servicing conditions cannot be overemphasized as well. Indeed, any changes in local pH and concentration can well be resulted from other multiple factors, such as the nature of electrolyte and the morphology of the catalyst surfaces, they all can affect the dynamic surface chemistry of catalysts and the OER trio. Therefore, an appropriate modeling of the pH and concentration gradients near the catalyst surfaces will be a key to correctly understand the dynamic surface chemistry of the OER catalysts. One shall expect that the combination of such models with surface kinetics models will become increasingly important in the dynamic surface chemistry of OER catalysts beyond catalyst design itself. Such a combination investigation can help direct the catalyst design to a furtherly comprehensive level.

The local pH environment around a catalyst surface also presents an obvious influence on the intermediate steps of OER,[^19^, ^72^] so the electrocatalytic activity and selectivity can be regulated by varying the electrolyte composition. Such effect was primarily discovered on OER in the seawater electrolyte, where chlorine evolution reaction (ClER) and hypochlorite formation are two competitive reactions of acidic and alkaline OER, respectively, such that the activity and selectivity of OER can be directly controlled by varying the (local) pH near the catalytic surface with additive ions or buffer.^[^ ^73^ ^]^ Compared to the sluggish 4e^−^ pathway of OER, only 2e^−^ involves into chloride chemistry on catalyst surface, leading to a faster kinetics at a smaller overpotential. Consequently, a commonly high potential window can be enabled by maximizing the thermodynamic potential difference of both two reactions, selecting OER pathways. When the bulk pH value is larger than 7.5, such a potential difference is even maximized up to 480 mV. Therefore, the selectivity of seawater splitting can be enhanced by upshifting the alkaline pH values. For example, high OER selectivity at the large current densities has been confirmed on the NiFe–LDH electrocatalyst by using borate buffered and 0.1 m KOH chloride‐containing electrolytes.^[^ ^74^ ^]^ Recently, our group has also demonstrated that a larger current density toward cathodic HER in the 0.1 m KOH seawater electrolyte can be obtained on the defective Ni single atom catalysts (SACs) compared to commercial Pt/C catalyst.^[^ ^75^ ^]^ It would be considerable value to in‐depth study whether the selectivity of OER is steered by (local) pH‐induced surface chemistry changes of catalyst at such high‐oxidation potentials, which can well be the fundamental origin. In addition, buffering halide ions into the electrolyte also contribute a key influence on the reconstruction reaction,^[^ ^76^ ^]^ although few studies have been reported.

In association with the local pH effect, the electrode morphology can have an impact on the dynamic surface chemistry and consequently OER activity of catalysts. A primary example has shown a sequence of OER activity being NiS (sugar cubes)> NiS (agglomerated stone particles) > NiS (apple shaped) in the strongly alkaline pH.^[^ ^77^ ^]^ The EIS analysis concludes that morphology varies the mass transportation to and from the catalyst, which influences the local pH and produces the different degree of surface reconstruction. In addition to that different morphology has quite different reconstruction kinetics at a given potential, the electrode morphology can also lead to difference in reconstruction reactions on the catalyst surface. As a verification, a couple of perovskite materials appear to be slow in reconstruction in the bulk phase, at even higher pH values than those of film phase.^[^ ^29^, ^78^ ^]^ Note that one should be mindful with the interpretation and comparison of experimental data of high‐surface‐area electrocatalysts on the dynamic surface chemistry, since the local pH effects may be significantly different compared with the bulk pH, leading to deviations on the dynamic surface chemistry.

The nature and concentration of electrolyte anions and cations present an obvious influence on the dynamic surface chemistry of OER catalysts, enabling an efficient control on OER performance beyond catalyst design. For example, a dynamic migration of fluorine anions leads to an enrichment in the catalyst surfaces (i.e., CoOOH) and eventually increases the hydrophilicity of surfaces, therefore accelerating the key process of surface chemistry and oxygen‐related intermediate adsorption.^[76b]^ In a glass‐ceramic (Ni_1.5_Sn@triMPO_4_) electrode made by embedding crystalline Ni_1.5_Sn nanoparticles into amorphous trimetallic phosphate (triMPO_4_) matrix, there is the leaching out of PO_4_^3−^ anions and subsequent exchanging with hydroxide anions in the OER process. Therefore, the dynamic surface chemistry is controlled by both electrolyte composition and surface terminations of catalysts, where it triggers favorable oxygen‐containing intermediate steps.^[^ ^79^ ^]^ Another rationality of the anion effect can be a lower local pH, compared with the situation without buffer or additives in the electrolyte, due to the competitivity of exotic anions with hydroxide anions.

The effects of cations on the surface chemistry of OER catalysts have been widely investigated, where they are generally interpreted in terms of metal diffusion between the catalyst surface and electrolyte, change in the surface reconstruction rate, generation of oxygen vacancies, alteration in the electronic structure of active sites, and consequently increasing the overall OER activity^[^ ^80^ ^]^. There is also possibility of changing the OER mechanism from adsorbate evolution mechanism (AEM) to lattice oxygen‐mediated (LOM) mechanism. (The details of these two mechanisms will be discussion in the computed section.) Furthermore, certain metal cations (i.e., iron^[^ ^81^ ^]^) can dynamically diffuse into the reconstructed surface during OER process, subsequently facilitating the enhanced OER kinetics. Certain metal cations can also aid the crystallization of reconstructed surfaces by forming stronger metal–oxygen bonds, leading to an enhanced catalytic stability.[^7^, ^82^]

## 5 Intrinsic Morphology and (Sub)Surface Atoms of Precatalysts

As has been discussed, there are several key factors of reaction and servicing conditions as well as precatalysts themselves including phase, composition, and pore/hollow structures, deeply influencing the dynamic surface chemistry. Here, we specifically devote our focus to the surface composition and morphology down to atomic level since they as an origin reason have an interactive influence on other aspects for reconstruction and vacancy dynamics. Specifically, Ni‐ or Co‐based precatalysts undergo a surface reconstruction into the active surface phase of Ni or Co oxyhydroxides, and can preserve the surface cations stoichiometry. In contrast, La‐based catalysts lead to high overpotentials and do not evolve into an active surface phase even after tens of hours of operation.^[^ ^83^ ^]^ In addition, the doping of some heteroatoms also can affect the surface reconstruction due to the modulation in electronic structure of multimetals in the catalysts. For instance, an incorporation of Fe in NiSe_2_ can adjust the redox ability of Ni and decrease the average valence state of Ni in the reconstructed NiOOH, meanwhile, increase the valence state of Fe, therefore providing more active sites.^[^ ^84^ ^]^ Moreover, the strong electron interactions between Fe, Ni, and Se can lead to an increase in electron possession of Se and enhanced ability of electron donating, which make it easier to be oxidized in alkaline solution. As a result, metal selenide materials are easier to form SeO_*x*_ and metal hydroxide by the surface reconstruction during the OER.

**Figure 7:**
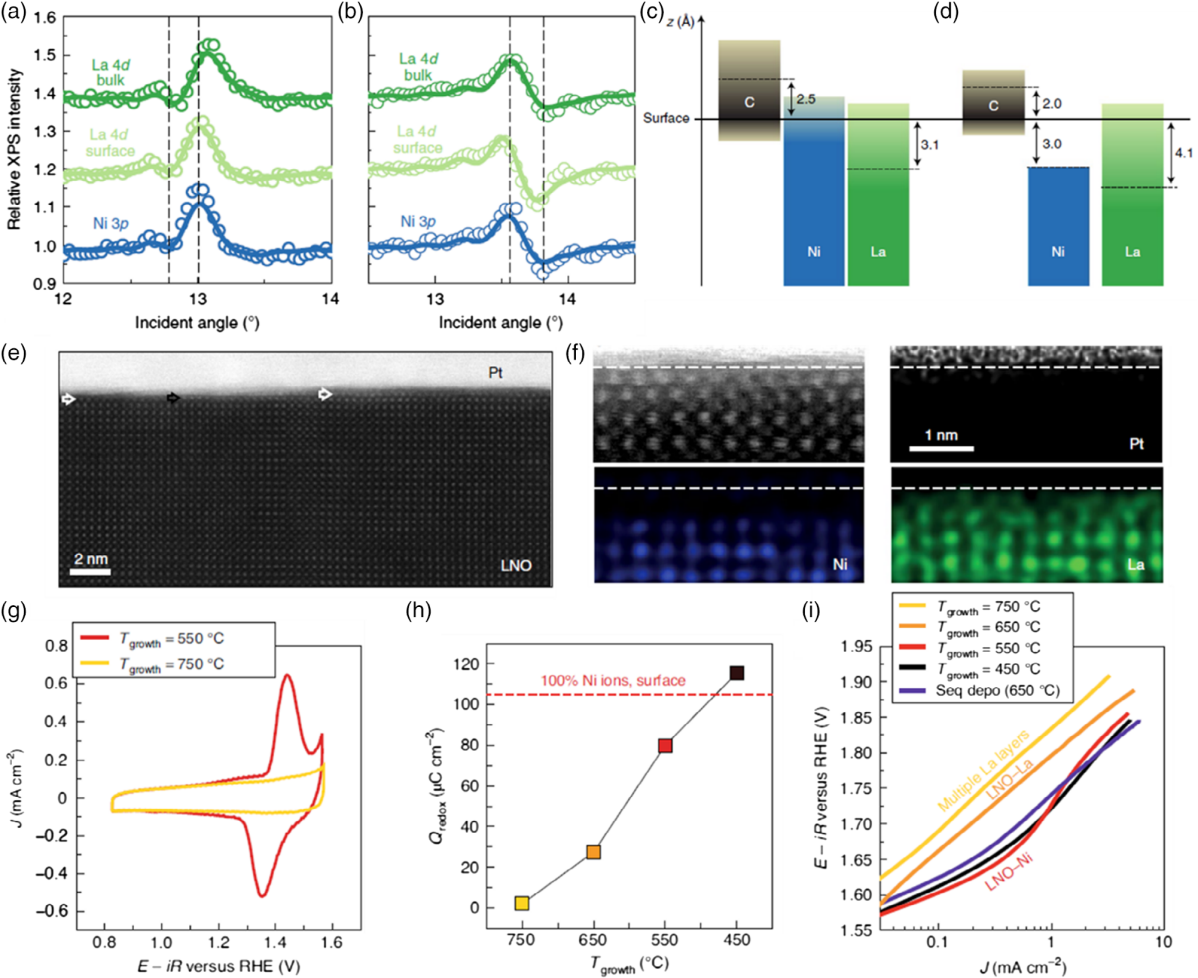
a,b) La 4d and Ni 3p SW–XPS rocking curves for LNO top layers deposited at 450 and 650 °C. Open circle data points and solid lines represent the measured intensities and best fitting results, respectively. c,d) Optimized models used to simulate the rocking curves in a (c) and b (d). e) STEM high‐angle annular dark‐field image for *T*_growth_ = 650 °C. f) High‐resolution STEM high‐angle annular dark‐field image and EDX spectroscopy distribution of Pt, Ni, and La for the same sample as in (e). g) Cyclic voltammetry at 500 mV s^−1^ for samples with low and high growth temperature. h) Redox charge of the Ni^2+^ to Ni^3+^ conversion during cyclic voltammetry. i) OER activity for different surface compositions in a Tafel‐like plot. a–i) Reproduced with permission.^[^ ^83^ ^]^ Copyright 2021, Springer Nature.

Recently, different atomic termination has been demonstrated to show distinct influence on the dynamic surface chemistry of catalysts in the OER process. Baeumer and co‐authors have studied the surface transformation behavior during OER driven by the electrochemical potential in atomically flat LaNiO_3_ (LNO) thin films.^[^ ^83^ ^]^ The surface composition apparently influences the surface transformation, and as a result, the OER activity. Two approaches were adopted to explore the effect by changing the surface termination, where one is to use sequential deposition of a single NiO*x* layer on the La‐terminated LNO surface, and the other is to vary the growth temperature during pulsed laser epitaxy of LNO thin films on (001)‐oriented Nb:SrTiO_3_ substrates. The surface and subsurface composition was then uncovered by using standing wave XPS (SW–XPS). **Figure** **7**a,b show the SW–XPS rocking curves for two samples derived from aforementioned two approaches, where the rocking curves for Ni 3p and La 4d surface were observable in phase as the growth temperature (*T*_growth_) rose to 450 °C, whereas they were out of phase at the *T*_growth_ of 650 °C, indicating that Ni was rich at the lower growth temperature, whereas La was rich at the higher growth temperature. A Ni‐free surface layer with a 3 Å thickness on the LNO film at the *T*_growth_ of 650 °C and a 3 Å thick Ni‐rich surface layer at the *T*_growth_ of 450 °C were obtained, respectively (Figure 7c–e). The STEM investigation also verified the aforementioned conclusion (Figure 7f), where at the *T*_growth_ of 450–550 °C, a Ni‐rich layer was obtained on the surface of LNO film. Although the sample grown at 650 °C displayed a mixture of LaO termination and a double LaO*x* layer on the surface, corresponding to the 3 Å Ni‐free layer. When the growth temperature rose to 750 °C, the La covered surface layers became even thicker. In this sense, adjusting the deposition temperature can selectively control the surface composition while keeping the bulk stoichiometry unchanged and the morphology comparably smooth. A striking difference in the pre‐OER regime was observed between surface Ni‐rich LNO (LNO–Ni) and La‐rich LNO (LNO–La), during CV as shown in Figure 7g. LNO–Ni demonstrated the characteristic redox peak at a potential of 1.4 V (vs RHE), whereas no obvious redox peak occurred in the thicker La‐covered LNO even after tens of CV cycles or 16 h of chronoamperometry at various potentials, revealing a surface reconstruction only existing in LNO–Ni. As the growth temperature rose, the surface coverage of Ni gradually decreased, suggesting that the magnitude of the redox wave decreased monotonically (Figure 7h), signifying a surface‐limited redox process. The OER activity decreased in subsequence of LNO–Ni, LNO–La, and thicker La‐covered LNO corresponding to the growth temperature of 550, 650, and 750 °C, respectively, as shown in Figure 7i, indicating a crucial effect of the surface composition on the reconstruction and OER activity.

In general, OER trio including activity, intermediate adsorption/desorption, and stability, predominantly depends on the nature of active sites present on the surface.^[^ ^85^ ^]^ Nanostructured and mesostructured catalysts can offer abundant active sites, realized by controlling the catalyst particle size, local structure, etc. Normally, a rough surface with high surface area inevitably exhibits improved OER performance in comparison with smooth surfaces of low surface area, which is typically ascribed to the increased population of undercoordinated sites. A small particle size of precatalyst can give rise to a rapid and deep surface reconstruction, and generate a small size of reconstructed catalyst proving a large active surface area. As the feature size decreases to the nanometer scale, these contributions are comparable to those of the surfaces. For instance, Zeng et al. have successfully modulated the *e*_g_ filling of cobalt ions to 1.2, which was regarded as an optimized value to obtain a high OER activity based on the Shao‐Horn's principle, by reducing the particle size to ≈80 nm.^[^ ^86^ ^]^ The resultant OER activity is higher than those of other‐sized samples as well as the bulk. The small nanoparticle size can effectively expose a higher density of Co cations (active sites) on the surface, facilitating the efficient spin‐state transition from low‐spin to high‐spin states of cobalt ions at the surface of the nanoparticles.

Two‐dimensional ultrathin nanosheets show a large surface area, expose more active sites, and enable several fascinating physical and chemical properties due to the dimensional confinement.^[^ ^87^ ^]^ Huang and co‐workers synthesized CoOOH nanosheets with a layer thickness of 1.4 nm by a 2D “atomic‐scale phase transformation” strategy derived from Co(OH)_2_ in which nearly all the metal ions can be exposed, therefore providing high mass active sites for the OER.^[^ ^88^ ^]^ The dimensional confinement in CoOOH ultrathin nanosheet can change the electronic structure to half‐metallic behavior (i.e., 52‐fold increase in electric conductivity in comparison to that of the bulk), facilitate the interfacial charge transfer, and facile electrochemical reactions to decrease the catalytic reaction barrier. Such 2D ultrathin nanosheets can be obtained by techniques, such as sonication, liquid‐phase exfoliation, etc.^[^ ^88^, ^89^ ^]^ For the layered materials such as LDH and MoS_2_, liquid‐phase exfoliation is an efficient method, where a prerequisite for the successful exfoliation is to enlarge the interlayer distance, resulting in weakening of the interaction between molecules in the layers and interlayers. Then, interlayer anion exchange can be proceeded by dipping the as‐prepared layered material into concentrated aqueous solutions of target anions.^[^ ^90^ ^]^ The increase in interlayer spacing after anion exchange allowed the delamination of the bulk LDHs to single‐layer nanosheets with higher surface area and more exposed active sites.

Similarly, porous and hollow structures can also expose abundant active sites, increase surface area, and accelerate the surface reconstruction. For example, Guo and co‐workers produced abundant oxygen vacancies by leaching out of sulfide in NiS_2_/CoS_2_ under an electrochemical condition.^[^ ^91^ ^]^ The NiS_2_/CoS_2_ electrocatalyst with abundant oxygen vacancies (NiS_2_/CoS_2_–O) exhibited much smaller mesopore size of ≈4 nm in diameter, compared to the bulk NiS_2_/CoS_2_ (major pore with a diameter of ≈40 nm), indicating a high‐porosity structure. Based on the abundant oxygen vacancies and porous structure, a more complete reconstruction in NiS_2_/CoS_2_–O was achieved. Hollow‐structured electrocatalysts have also been widely used for OER because they can provide a high active surface area for the accelerating reconstruction reaction.^[^ ^92^ ^]^ The metal organic frameworks (MOFs) as the precursors or templates are normally used to design the hollow structure by an annealing method at a high temperature. Lou and co‐authors synthesized a Co_3_O_4_/Co–Fe oxide double‐shelled hollow nanoboxes via anion exchange reaction and subsequent annealing treatment based on the zeolite imidazole framework (ZIF) nanocubes.^[^ ^92^ ^]^ A larger reconstruction area was enabled for providing more catalytic sites due to the increased the electrode/electrolyte contact area from the double‐shelled hollow structure. Yang et al. fabricated cobalt‐molybdenum nitride materials with hollow 3D structures and 2D nanosheets based on the MOF (ZIF‐67 and Mo‐MOF), which exhibit high OER activities.^[^ ^93^ ^]^ It was found that the sample structure and morphology strongly rely on the ligand–metal interactions of MOF, and deeply determine the lower reconstruction potential and well‐confined surface configurations after reconstruction, contributing to the improved OER activity. Presently, one of the major challenges of porous or hollow catalysts for electrocatalysis is that the very small pore size impedes the effective mass transport to the active sites and the diffusion of products, leading to lower reconstruction area and rate.^[^ ^76a^ ^]^ The accumulation of product gas can also block the dynamic surface chemistry process (i.e., deeper reconstruction and faster vacancy filling) and then result in the structural collapse of the catalysts. So, the rational design of pore size and hollow structure is very important to deepen the understanding of the interaction between dynamic surface chemistry and catalytic activity.

## 6 In Situ Experimental Tracking of Dynamic Surfaces

**Figure 8:**
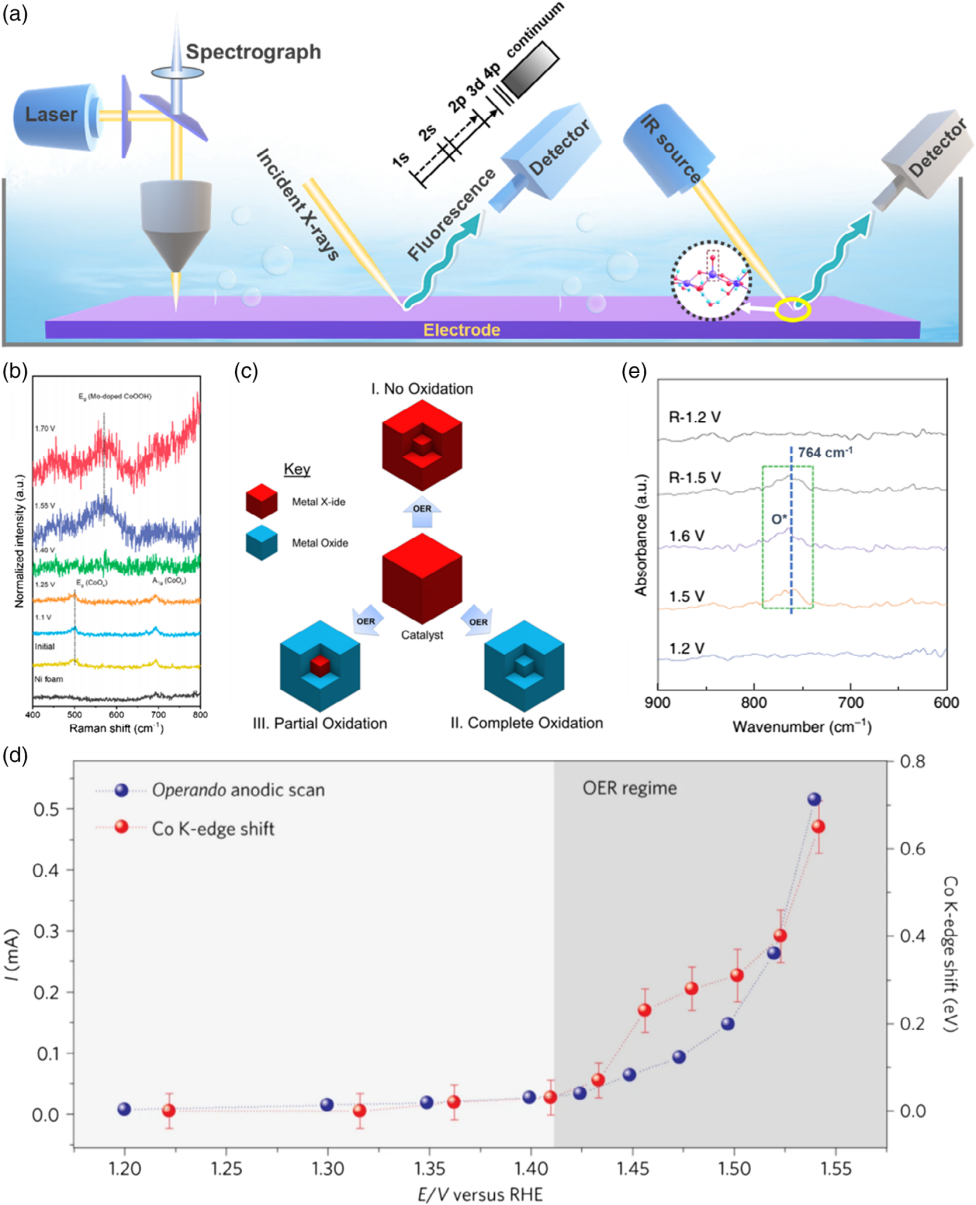
a) Schematic image of in situ spectroelectrochemical techniques. b) Raman spectra for understanding the reconstruction by probing the phase transformation of Co–Mo_2_C catalyst in the reaction process. Reproduced with permission.^[^ ^16^ ^]^ Copyright 2020, American Chemical Society. c) An overview of possible reaction pathways for a hypothetical metal X‐ide catalyst during water oxidation. Reproduced with permission.^[^ ^65^ ^]^ Copyright 2018, American Chemical Society. d) Operando XAFS for investigating the dynamic change of bond and coordination by monitoring current and shift of the Co K‐edge position (taken at the position of half the edge step height) recorded during an anodic polarization of the BSCF–FS electrode in the spectroelectrochemical flow cell. Reproduced with permission.^[15a]^ Copyright 2017, Springer Nature. e) Operando SR–FTIR spectroscopy measurements for understanding the mechanism by observation of adsorbed oxygen intermediate on Ru–N–C during the acid OER. Reproduced under the terms of the CC‐BY 4.0 license.^[^ ^95^ ^]^ Copyright 2019, The Authors, published by Springer Nature.

Several in situ spectrometric techniques, such as Raman, XAS spectroscopies, and FT–IR have been used, aiming at identifying the dynamic surface chemistry, including the surface reconfigurations and vacancy dynamics as well as surface‐adsorbed intermediates, where the observation or evolution is made as a function of several reaction parameters such as electrode potential, external field, and electrolyte composition. An overview of typical spectroelectrochemical techniques used for studies of dynamic surface chemistry on catalysts is shown in **Figure** **8**a.

In the dynamic surface chemistry, as a result of the reconstruction reaction, there are a range of changes in catalyst surface change, for example, as a function of potential, for which in situ Raman assisted with ex situ characterizations (i.e. XRD, XPS, and XAS) can be used to monitor the phase, composition, and chemical bond dynamics. Electrochemical oxidation of metal X‐ides is commonly observed at a potential range of about 1.2–1.5 V versus RHE in the alkaline and neutral electrolytes probed by CV, linear sweep voltammetry (LSV), and chronopotentiometry (CP), whereas acidic OERs are largely catalyzed by oxides as reported and therefore rarely refers to electrochemical oxidation activation. Electrochemical oxidation has been observed to stabilize or stop at a certain time and a given potential/potential cycling. By choosing a microzone in the sample on the catalyst surfaces by optical spectroscopy in a cell, Raman scattering enables one to study the vibrational mode changes of chemical bonds. Taking Co–Mo_2_C heterostructure nanoparticles as an example,^[^ ^16^ ^]^ in situ Raman tests were utilized to trace the phase transformation and surface reconstruction on the catalyst surface, as a function of applied potential (Figure 8b). Repeated LSV tests at different potential intervals were performed to ensure the stable phase and surface configurations. Prior to the potential applied up to 1.25 V, the catalyst only showed a slight degree of oxidation on the surfaces. When the potential exceeded 1.4 V, all the bands of the catalyst disappeared, suggesting a transformation of the surface oxide layer into γ‐CoOOH where Mo atoms gathered on the topmost surfaces. Finally, Mo‐enriched γ‐CoOOH acts as active species for OER. Such a potential‐dependent phase transformation and surface reconstruction has been revealed by in situ Raman investigations in several catalysts.^[^ ^1^, ^15^, ^79^ ^]^ As a result, on basis of these real‐time changes in composition of the OER catalysts and the nature of the catalytic site, most metal X‐ides can be referred to one of the three broad categories (Figure 8c). The first one is X‐ides themselves, where there are largely unchanged in surface structure even under rather rigorous OER conditions. The second one states that X‐ides are completely converted into amorphous metal oxide/(oxy)hydroxides upon the OER. In general, such a transformation involves with ultrasmall features of OER precatalysts and/or there is existence of some exotic elements in the precatalysts or electrolyte. The final yet most populated one involves the formation of a core–shell structure where X‐ides core is covered by an amorphous oxide/(oxy)hydroxide shell acting as the active species.

Upon the phase change, the composition of the catalyst can be identified by using in situ/ex situ Raman tests assisted with other advanced characterizations. In situ synchrotron XAS can be performed to detect the changes in local bond and environments around the active sites. Benefiting from the high intensity and monochromaticity, and tunable X‐ray wavelength of synchrotron radiation light source, the synchrotron radiation XAS can help identify the transitions from core electronic states of a metal to the excited electronic states lowest unoccupied molecular orbital (LUMO) and the continuum with high resolution; the former is known as the XANES which provides the key electronic structure and symmetry of the metal site, and the latter as the extended XAFS (EXAFS), which studies the fine structure in the absorption at energies greater than the threshold for electron release and it gives the numbers, types, and distances to ligands and neighboring atoms from the absorbing element.^[62b]^ Moreover, in situ synchrotron‐based XAS technique is cable of delivering understandings on the interfacial electrochemistry, and it has recently been used to gain insight into the chemical state changes of the active transition metal species on oxyhydroxide catalysts for OER. For example, Fabbri, Pertoso, and their co‐authors have combined a scalable cutting‐edge synthesis method with time‐resolved XAS measurements to capture the dynamic local electronic and geometric structure of highly active OER perovskite nanocatalysts (Ba_0.5_Sr_0.5_Co_0.8_Fe_0.2_O_3‐δ_ nanopowder, named as BSCF) under rather realistic operando OER conditions^[15a]^ (Figure 8d). Unlike that was discussed for the aforementioned Co‐Mo_2_C example, Co K‐edge XANES spectra confirm that change cannot be detected by simply soaking BSCF in the 0.1 m KOH electrolyte, when one increases the applied potential from 1.2 to 1.425 V. A further increase in the applied potential exceeding 1.425 V resulted in a upshift of the Co K‐edge to higher energy, suggesting an improved Co oxidation state. When progressively increasing the potential above 1.55 V, a higher oxidation degree was achieved of Co cations. However, upon recovering of the applied potential from 1.55 to 1.2 V, it cannot generate a significant change of Co oxidation, indicating that such reconstruction is mostly an irreversible process. Interestingly, unlike Co, Fe cations remain unchanged during the overall OER process, demonstrating that Fe cations can hardly involve in the reconstruction reaction. Their results suggest that the electronic structures of the active elements in the OER catalysts can drastically change, after the onset of OER under the operando OER conditions. Therefore, it could be a misleading by simply correlating the OER activity to certain ex situ physical properties, and by comparing the structural and performance changes before and after the OER process.

Variations in local coordination environment of active sites under operando conditions can be monitored by operando EXAFS spectroscopy. For example, the Fourier‐transformed (FT) k^3^‐weighted EXAFS spectra of BSCF–FS in the aforementioned example have been studied in situ at different applied potentials. In addition to the identification of active CoOOH and FeOOH, FT–EXAFS results conducted at Co K‐edge concluded that a large population of oxygen vacancies were generated in the BSCF–FS sample, when one gradually increased the potential. Therefore, one can conclude that vacancy dynamics is mostly potential dependent. Indeed, both the vacancy dynamics and reaction mechanisms have been investigated by in situ isotope labeling electrochemical mass spectrometry (EC–MS) and nuclear magnetic resonance (NMR). The ^18^O‐labeled perovskites were modeled experimentally to directly monitor the LOM by detecting O_2_ products with EC‐MS.^[^ ^37^ ^]^ Furthermore, in situ EC–MS was applied to detect whether oxygen ions were involved in the OER process of NiFe‐based nanocatalysts, which are regarded as a promising OER catalyst in the alkaline electrolytes.^[^ ^94^ ^]^ Different from the perovskites, the oxygen ions in NiFe‐based catalysts remained basically unchanged. This information is useful in the development of better OER catalysts as it significantly shifts existing paradigms. These in situ spectroscopy investigations help strengthen the operando understanding on dynamic surface chemistry of OER catalysts.

Operando FT–IR has been developed to detect the changes in chemical bonds between active metal sites and oxygen‐containing intermediates in OER process. For example, the dynamic adsorption of single oxygen atom on atomically dispersed Ru_1_–N_4_ site anchored on nitrogen–carbon support (Ru–N–C) was investigated by operando FT–IR under the acidic operation environment (Figure 8e).^[^ ^95^ ^]^ At the first sight, there is no obvious absorption band that can be discerned over the low‐vibration frequency region of 900–600 cm^−1^ for the catalyst at 1.2 V versus RHE or lower potentials. When higher potentials of 1.5 and 1.6 V versus RHE were applied, however, a new prominent absorption band appeared at ≈764 cm^−1^ in the FTIR spectra, suggesting the emergence of a crucial intermediate in the OER process. Moreover, when reversing the potential from 1.6 to 1.2 V versus RHE, the new absorption band had gradually disappeared, indicating the reversible adsorption and desorption of intermediate. To clarify the origin of this vibrational absorption band, DFT was applied to calculate the different vibrational absorption bands in several possible configurations. The wavenumber of vibrational absorption band for single oxygen adsorption in the O–Ru_1_–N_4_ configuration is quite close to the new peak position observed in the operando FTIR spectra, suggesting that the intermediate species come from the single oxygen adsorption. Therefore, by the one‐by‐one characterizations from in situ Raman, XAS, and EC–MS to FTIR, one can make key understandings on the surface reconstruction and vacancy dynamics, changes in local bond and environments of active sites, and intermediate adsorption/desorption dynamics.

Although in situ spectrometric techniques have been widely developed to monitor the dynamic surface chemistry of catalysts and catalysis, they mainly provide the bulk/average information of those processes on catalyst surfaces and/or catalyst–electrolyte interfaces. To real‐time observe the local variations of dynamic surface chemistry, it is critically important for developing the operando imaging techniques (i.e., environmental and cryo‐transmission electron microscopy/atomic force microscopy). However, it faces huge challenges since these dynamic processes are operated in liquid‐based environments which is highly unfavorable for imaging techniques commonly sensitive to aqueous conditions. Therefore, current imaging techniques are mainly applied to in situ see the morphological evolution but rarely operate at the atomic precision, letting the local observation of dynamic surface chemistry alone.

## 7 Computational Approaches to Dynamic Surfaces

**Figure 9:**
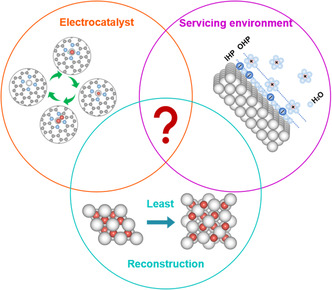
An overview of computational emphasis comparison among the electrocatalyst surface, servicing environment, and reconstruction process.

Substantial contributions have been made by modeling and simulations to understanding and optimizing the electrochemical interfaces. Among the several common theoretical methods, DFT is a very popular computational method in the electrocatalysis field, which is a first‐principles tool that can be used to make key understandings on catalytic processes and identify promising candidate materials through examinations of the kinetic and thermodynamic aspects.^[^ ^96^ ^]^ As shown in **Figure** **9**, DFT calculations have been widely applied to electrocatalysts, including their formation, structure, and predicted performance, where the importance of static modeling concepts is obviously witnessed. There is a detailed review that has been published more recently on the fundamentals of OER.^[^ ^48^ ^]^ By comparison, the actual electrocatalysis process has been less studied by DFT calculations, where dynamic descriptors are largely lacked. Indeed, there are few computational simulations on the true dynamic surfaces in the actual electrocatalysis process. Similarly, there is little computation study on the surface reconstruction and surface dynamic chemistry. There shall also be investigations into the interactions among the three key aspects, namely electrocatalysts, electrocatalysis and surface reconstruction, which have not been comprehensively considered in DFT calculations. Therefore, there remains a huge gap between the computational studies and experimental observations.

However, DFT simulations alone cannot thoroughly and clearly describe the electrocatalysis process, therefore, a multiscale modeling and simulation approach is required to connect the different efforts and to correlate the modeling and simulation results directly to the experiments. Molecular dynamics (MD) simulation is a method capable of detailed microscopic modeling on the molecular scales and allowing to follow the movement of individual atoms/molecules. Kinetic Monte Carlo (kMC) simulations can be applied to any system describable as a set of minima of a potential‐energy surface, the evolution of which will then be regarded as hops from one minimum to a neighboring one. State‐space modeling (SSM) is known in control theory to simulate complex, interdepending systems. Sinha et al.^[^ ^97^ ^]^ presented a multiscale computational model to elucidate the mechanism of the OER at the hematite–water interface. The model connected the thermodynamics and kinetics of elementary charge transfer reactions in OER to kinetics of OER at laboratory length and timescales. They coupled DFT and DFT‐based molecular dynamics (DFT–MD) simulations with solvent effects at an atomistic level with kMC simulations at a coarse‐grained level in the multiscale model. The multiscale model demonstrates the effect of explicitly examining the interaction of water with the electrode surface via direct adsorption. However, at present, the multiscale has not been widely applied in the surface reconstruction process during OER, therefore, more and further researches need to be done in the multiscale modeling and simulation.

It is widely acknowledged, in both theoretical consideration and experimental observation, that OER proceeds by the catalytic species in two mechanisms: AEM and LOM, as mentioned before. For AEM, four concerted proton–electron transfer (CPET) reactions are correlated to the OER on catalytic sites. The first step is the adsorption of OH^−^ on a catalytic site to form HO* species. Subsequently, the HO* species deprotonate to evolve into O*. And then, O* further reacts with another OH^−^ to generate the HOO* intermediate. Finally, the deprotonation of HOO* results in the evolution of O_2_ and releases the active sites. Computational hydrogen electrode (CHE) has been commonly applied as the reference potential to combine with DFT‐calculated adsorption energies of the intermediates for gaining insights into the coverage of surface species at certain pH and potentials, according to the surface Pourbaix diagrams and the reaction free energies (Δ*G*) of intermediate steps.^[^ ^98^ ^]^ The most positive Δ*G* gives not only the overpotential but also potential‐determining step (PDS), therefore determining the catalytic performance. Moreover, since the adsorption energies of adjacent intermediates are commonly linearly correlated on one specific active site, the scaling relation can be described by the difference in their Δ*G*. Specifically, a constant difference (Δ*G*_HOO*_‐Δ*G*_HO*_) have been confirmed to be 3.2–0.2 eV on either metal or oxide surfaces, such that they can be directly calculated by each other when one is known.^[^ ^48^ ^]^ By properly comprehending the aforementioned considerations together with the adsorption energy difference on PDS in most OER catalysts, the adsorption energies difference (Δ*G*_O*_‐Δ*G*_HO*_) is widely accepted as a key descriptor of OER activity, where the overpotential is expressed as *η* ^OER^ = {max[(Δ*G*_O*_‐Δ*G*_HO*_), 3.2 eV‐(Δ*G*_O*_‐Δ*G*_HO*_)]/e. The activation and optimization of catalytic sites are mainly dependent on regulating the value of Δ*G*_O*_‐Δ*G*_HO*_ by engineering the electronic structure of active sites, although some other descriptors, such as *e*_g_ orbital occupancy,^[^ ^99^ ^]^ metal–oxygen covalency,^[^ ^100^ ^]^ and etc., have been also adopted to identify the OER activity.

Following the discussion on the important roles played by the reaction and catalytic servicing conditions, it is clear that CHE‐based models are not pH dependent and therefore not applied to express the decoupled proton–electron transfer (DPET) steps, although these reaction steps have been experimentally observed on metal and oxide electrocatalysts through the pH effects on the RHE scale. More importantly, the reaction and servicing conditions can contribute to determining the dynamic behaviors of catalyst surfaces, and furtherly the OER activity. Therefore, these static descriptors can be only used to screen the OER catalysts, and it will be difficult for them to be directly correlated to the active origin and dynamic fundamentals. As a result, dynamic correction items and the variation in catalytic sites should be considered.

The scaling relations can indeed help reduce the complexity of simultaneous DFT‐based screening of catalysts by the Sabatier‐type activity plot. It is hard to not only identify the dynamic influence of applied potential on the OER activity as discussed before but also rarely deals with the dynamic changes of active sites. Due to the constraints in association with obtaining an optimal OER electrocatalyst posed by scaling relations, numerous studies have called upon breaking the scaling relations between the OER intermediates to substantially boost OER activity, which is also available for most complex catalytic reactions. For example, the reversible insertion of hydrated K^+^ inside the open crystallographic structure of α‐Li_1_IrO_3_ enables the phase transformation of the catalysts into a birnessite structure.^[^ ^101^ ^]^ DFT calculations have confirmed that this dynamic conversion induces a change in the adsorption energies of the OER intermediates and break the linear correlation of the energies of the intermediates. Although the boosted OER activity was simply assigned to one specific active site, rather than the synergistic functions of multiple active sites, this finding provides a rather universal explanation to several other layered compounds with the open crystallographic structure and therefore facilitates the rational understanding of previous observations related to the effect of alkaline ions on the dynamic behavior of catalysts and their OER kinetics.

Another example of breaking scaling relation is the individual balance of different intermediate adsorption/desorption on different active sites in one catalyst entity. Our group have confirmed that anchoring HER‐selective Mo single atom sites around the nitrogen reduction reaction (NRR)‐selective Mo_2_C species can synchronize the selectivity and activity of NRR by prohibiting HER on the Mo_2_C and synergistic intermediate steps on these two active species.^[^ ^102^ ^]^ This work contributes a fundamental concept of circumventing scaling relations in complex catalytic reactions, such as NRR, OER, and CO_2_ RR. Indeed, there have been rigorous research efforts on the rationality of breaking scaling relations. For example, a single AEM for OER is applied to draw the scaling relation‐based volcano plot for all the catalysts and their facets, ignoring other uncertainties, such as dynamic changes of active sites, crystal facet evolution, strain effect, and etc. A more recent study has further suggested that breaking the scaling relations is necessary but insufficient condition for a breakthrough of catalytic performance, since the activity is not only both material and structure sensitive but also rather related to the reaction and servicing conditions.^[^ ^103^ ^]^

Adsorbate solvation or surface terminations when soaking a catalyst into the electrolyte could play a key role on the dynamic surface chemistry of catalyst, although it has not been sufficiently considered in the DFT‐based models. For example, the effect of solvent water on the AEM of IrO_2_ (110) has been found in which water can remarkably alter the geometry of adsorbed OER intermediates (i.e., HO* and HOO*) and switch the interaction with surfaces to that with the water bilayer.^[^ ^104^ ^]^ Moreover, increasing the water layer can hardly affect the adsorbate binding energies, suggesting that it is enough for applying a single water layer to describe the effect. Although the solvation has been widely considered as a constant value to correct the calculated adsorption energies in the vacuum, it was also recently found to be strongly dependent on the geometry and chemical nature of the surface sites. Indeed, our recent work has experimentally confirmed that a variation in the surface termination of holey 2D Mo_2_C catalyst from nitrogen to oxygen can help optimize the adsorption energies of the intermediates and therefore improve the intrinsic OER activity of surface sites.^[10a]^ Apart from the solvation effect, ion effect in the different electrolytes could also impact the adsorption energies of intermediates which requires a careful consideration into DFT calculations.

In contrast, two neighboring metal sites can activate the LOM, where two HO* first deprotonate into two metal‐oxo species, and then directly couple to form the O—O bond instead of combining with water or another OH^−^ to generate HOO* for directly releasing O_2_ to form two metal vacancies that are subsequently occupied by OH^−^. One can see that HOO* does not involve into LOM, such that converting AEM on a single site to LOM on two metallic sites can enable a simple breaking of scaling relation between HO* and HOO*.^[^ ^105^ ^]^ However, this process requires a large activation barrier, and litter progress has been achieved in the past few decades. In more recent years, several reputed groups have made substantial progress on activating LOM in several catalysts, including perovskites,^[^ ^100^ ^]^ binary,^[22c]^ and trinary metal oxides^[^ ^106^ ^]^ as well as LDHs^[^ ^107^ ^]^ through tuning the metal–oxygen covalency. Moreover, since metal vacancy dynamics is clear in the LOM, it is urgently required to develop dynamic vacancy‐related descriptors to evaluate the OER activity and find an optimal catalyst with lower overpotential. In addition, the effect of correction items on the metal vacancy formation and filling should also be considered.

Although substantial progress of simulation works has been made with OER, considerable challenges remain on an accurate screening of OER catalysts and their dynamic nature during the actual process. A proper understandings and thorough investigations into the dynamic surface chemistry are required, by developing the related simulation methods/descriptors, which shall be closely correlated to experimental environments. With properly incorporated features of real OER interfaces, one shall focus future efforts on the surface dynamics, as affected by the (local) pH and applied potential, where is need for an understanding on scaling relations, and how and when to break/avoid it. In addition, proper catalytic models are to be established, as well as simulations on the interaction among different factors, which is responsible for the dynamic change from precatalysts to real catalysts.

## 8 Future and Perspectives

In the past couple decades, and in particular the past few years, substantial progress has been made for the new discovery, monitoring of dynamic progresses on catalyst surfaces in the reaction and servicing environments, and their effects on the overall OER performance and efficiency. An optimization of the reconstruction process can be targeted by tuning the catalytic servicing condition and lowering the applied potential, which is also largely correlated to the precatalyst design, including composition, phase(s), morphology, and structure regulation. The breaking in scaling relation between HO* and HOO* is largely dependent on the optimization of electronic structures of active sites and/or their coordination environment (typically, e.g., introducing dual‐metal sites or metal‐oxo species). Therefore, a proper regulation of the reconstruction can help facilitate the generation of positive active species and optimization of catalytic performance, where there is a need for deepening the understanding on authentic active sites and in the dynamic processes.

The actual OER process is associated with a rather complicated dynamic surface chemistry and reconstruction, in addition to the reversible catalytic process (i.e., the vacancy dynamics, adsorption and desorption of reaction species, and release of products) of the catalyst. Some of these processes have been widely investigated and evidenced by both advanced ex situ, in situ, and operando characterizations as well as theoretical simulations, although some of the exact mechanisms and driving forces are still not completely well understood. For example, developing a truly meaningful theoretical calculation method for systematically capturing the entire dynamic process starting from the precatalyst to actual catalyst is still highly challenging. There are obvious competitions among the different dynamic processes of catalytic surface and OER, reversible, and irreversible reconstruction, where the efforts shall be directed toward enhancing the ultimate OER performance. The dynamic behavior of metal species (metal dissolution and vacancy formation/filling) has been revealed to be related to both precatalysts and their catalytic servicing environment, and eventually determining the performance of an underlying catalyst. For example, there is large set of precatalysts and their servicing environment conditions, such as the (sub)surface atomic structure, phases, morphology of the catalysts, and the reaction and servicing conditions (e.g., applied potential, local pH, ion transportation, and etc.), which shall be synchronously tuned. To this end, it is highly desired yet challenging to comprehend all these different aspects in both experimental and computational models, instead of a simple collection of individual/single effect from a system perspective.

**Figure 10:**
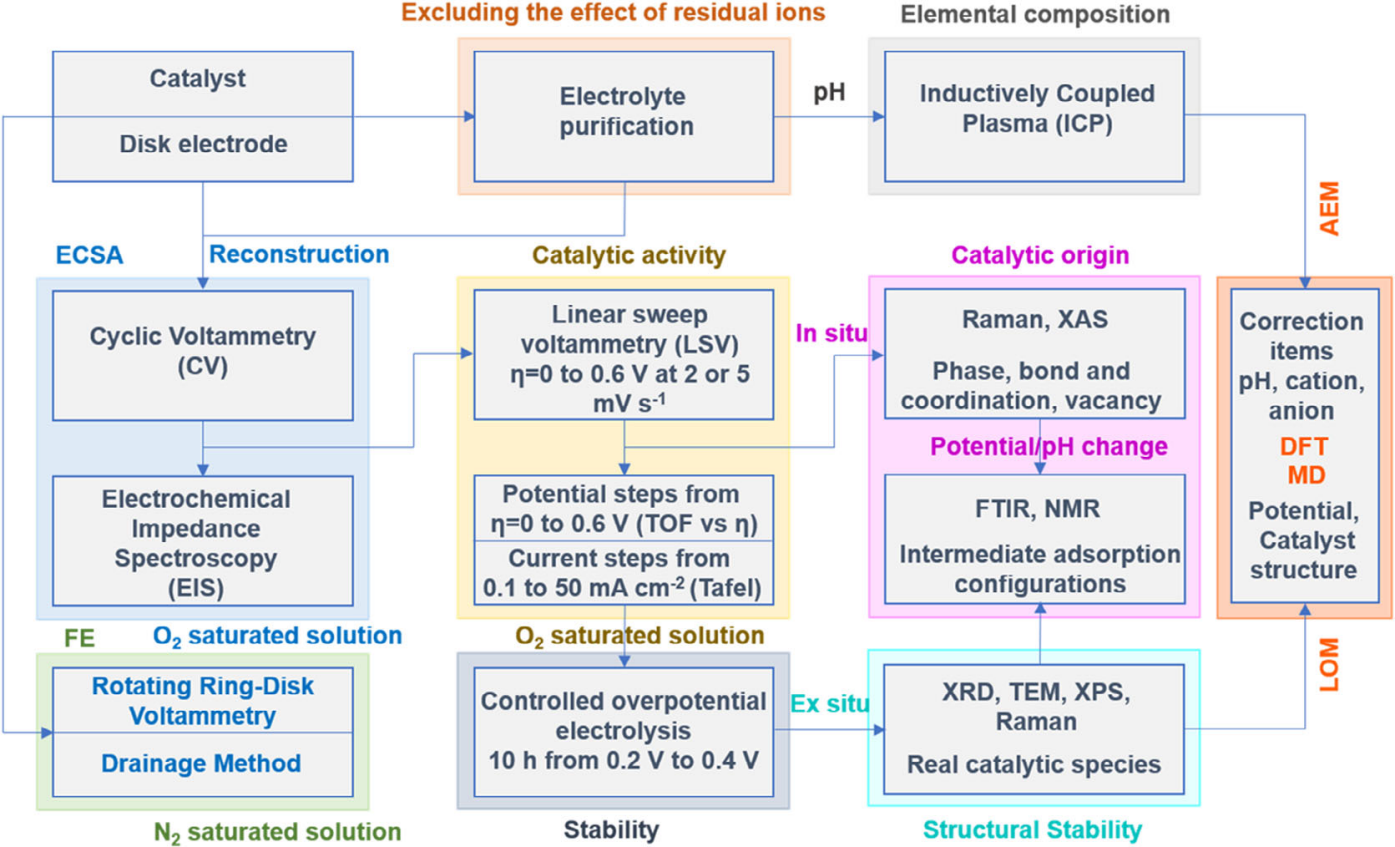
A brief frame of recommended protocols for electrochemical evaluation, structural characterizations, and mechanism understanding in OER.

Currently, although there are several advanced in situ and operando techniques that have been developed to detect and study surface chemistry changes of the OER electrocatalysts, an operando imaging of the dynamic OER intermediates and catalytic surfaces is rather limited in actual studies. Moreover, there is a general lack in the systematic considerations toward the competitiveness of different OER mechanisms in association with the dynamic changes of active sites and reconstruction. Therefore, there is requirement for development of high resolution, real‐time characterizations with ultrafast‐time response, to observe the truly dynamic processes of both OER and catalyst surfaces. To paint an overall picture of the fundamental principles, critical factors involved, and research methodologies on the dynamic surface chemistry, it would also require a more rigorous standardization of the experimental setups and procedures, where a proposed framework (**Figure** **10**) would be useful. Moreover, certain dedicated efforts shall be directed to the understanding of actual electrocatalysis process beyond what happens at the catalyst surface. For this purpose, multiscale computational modeling approaches encompassing all these aspects would be of value. With deep understandings as have been discussed/suggested in this overview, one would see that the future research should definitely go beyond the static electrocatalysts/electrocatalysis, as the chemistry dynamics of active sites on surface/subsurface matter the most.

A proper multiscale modeling and simulation approach requires to be explored for describing and understanding the surface reconstruction process during OER. Several simulations can be connected to uncover mechanisms at different levels. First, at atomistic level, DFT will be used to simulate the reactions at the electrode–electrolyte interface, which involve the calculation of the Gibbs free energies of each reaction step, transition states, activation energies, and rate constants. Second, at the molecular level, reactive force field molecular dynamics (ReaxFF) can be adopted to simulate the restructuring from the surface of catalysts during OER. DFT calculated data will be used to train a suitable reactive force field for the given system. The restructuring induced changes in catalytic activities can be further determined by DFT calculations. Third, kMC method can be utilized to simulate multiple reactions at the interfaces (i.e., adsorption/desorption, diffusion, and reaction). DFT and MD simulations are able to confirm the rate constants for these processes and the rate‐limiting reaction step, charge transport, and coverage dependence. Finally, SSM method can be used for simulating electrochemical data that can be directly compared to the experiments. The inputs of SSM are calculated by the other levels of methods as mentioned earlier. Using a proper optimization procedure with experimental data, the input data can then be optimized and kinetic parameters be determined. Then, the validation and verification will be done based on the matching with experimental data measured under diverse operating conditions.

## Conflict of Interest

The authors declare no conflict of interest.
